# FLT3L governs the development of partially overlapping hematopoietic lineages in humans and mice

**DOI:** 10.1016/j.cell.2024.04.009

**Published:** 2024-05-03

**Authors:** Mana Momenilandi, Romain Lévy, Steicy Sobrino, Jingwei Li, Chantal Lagresle-Peyrou, Hossein Esmaeilzadeh, Antoine Fayand, Corentin Le Floc’h, Antoine Guérin, Erika Della Mina, Debra Shearer, Ottavia M. Delmonte, Ahmad Yatim, Kevin Mulder, Mathieu Mancini, Darawan Rinchai, Adeline Denis, Anna-Lena Neehus, Karla Balogh, Sarah Brendle, Hassan Rokni-Zadeh, Majid Changi-Ashtiani, Yoann Seeleuthner, Caroline Deswarte, Boris Bessot, Cassandre Cremades, Marie Materna, Axel Cederholm, Masato Ogishi, Quentin Philippot, Omer Beganovic, Mania Ackermann, Margareta Wuyts, Taushif Khan, Sébastien Fouéré, Florian Herms, Johan Chanal, Boaz Palterer, Julie Bruneau, Thierry Molina, Stéphanie Leclerc-Mercier, Jean-Luc Prétet, Leila Youssefian, Hassan Vahidnezhad, Nima Parvaneh, Kristl G. Claeys, Rik Schrijvers, Marine Luka, Philippe Pérot, Jacques Fourgeaud, Céline Nourrisson, Philippe Poirier, Emmanuelle Jouanguy, Stéphanie Boisson-Dupuis, Jacinta Bustamante, Luigi D. Notarangelo, Neil Christensen, Nils Landegren, Laurent Abel, Nico Marr, Emmanuelle Six, David Langlais, Tim Waterboer, Florent Ginhoux, Cindy S. Ma, Stuart G Tangye, Isabelle Meyts, Nico Lachmann, Jiafen Hu, Mohammad Shahrooei, Xavier Bossuyt, Jean-Laurent Casanova, Vivien Béziat

**Affiliations:** 1. Laboratory of Human Genetics of Infectious Diseases, Necker Branch, INSERM UMR 1163, Necker Hospital for Sick Children, Paris, France; 2. Paris Cité University, Imagine Institute, Paris, France; 3. Pediatric Hematology-Immunology and Rheumatology Unit, Necker Hospital for Sick Children, AP-HP, Paris, France; 4. Laboratory of Chromatin and Gene Regulation During Development, Paris Cité University, UMR1163 INSERM, Imagine Institute, Paris, France; 5. Laboratory of Human Lymphohematopoiesis, INSERM, Imagine Institute, Paris, France; 6. Jake Gittlen Laboratories for Cancer Research, Pennsylvania State University College of Medicine, Hershey, PA, USA; 7. Department of Pathology and Laboratory Medicine, Pennsylvania State University College of Medicine, Hershey, USA; 8. Biotherapy Clinical Investigation Center, Groupe Hospitalier Universitaire Ouest, AP-HP, INSERM, Paris, France; 9. Allergy Research Center, Shiraz University of Medical Sciences, Shiraz, Iran; 10. Department of Allergy and Clinical Immunology, Namazi Hospital, Shiraz University of Medical Sciences, Shiraz, Iran; 11. Sorbonne University, AP-HP, Tenon Hospital, Department of Internal Medicine, Paris, France; 12. Garvan Institute of Medical Research, Darlinghurst, NSW, Australia; 13. St. Vincent’s Clinical School, Faculty of Medicine, University of NSW, Sydney, NSW, Australia; 14. Laboratory of Clinical Immunology and Microbiology, National Institute of Allergy and Infectious Diseases, National Institutes of Health, Bethesda, MD, USA; 15. St. Giles Laboratory of Human Genetics of Infectious Diseases, Rockefeller Branch, Rockefeller University, New York, NY, USA; 16. Gustave Roussy Cancer Campus, Villejuif, France; 17. Paris-Saclay University, Ile-de-France, France; 18. Dahdaleh Institute of Genomic Medicine, McGill University, Montreal, Quebec, Canada; 19. Department of Microbiology and Immunology, McGill University, Montreal, Quebec, Canada; 20. Department of Microbiology and Immunology, Pennsylvania State University College of Medicine, Hershey, PA, USA; 21. Department of Medical Biotechnology, School of Medicine, Zanjan University of Medical Sciences (ZUMS), Zanjan, Iran; 22. School of Mathematics, Institute for Research in Fundamental Sciences (IPM), Tehran, Iran; 23. Science for Life Laboratory, Department of Medical Biochemistry and Microbiology, Uppsala University, Uppsala, Sweden; 24. Laboratoire d’Onco-hématologie, Necker Hospital for Sick Children, AP-HP, Paris, France; 25. Hannover Medical School, Department of Pediatric Pulmonology, Allergology and Neonatology, Hannover, Germany; 26. Hannover Medical School, Cluster of Excellence RESIST (EXC 2155), Hannover, Germany; 27. Fraunhofer Institute for Toxicology and Experimental Medicine ITEM; 28. Department of Microbiology and Immunology, Clinical and Diagnostic Immunology, KU Leuven, Leuven, Belgium; 29. Research Branch, Sidra Medicine, Doha, Qatar; 30. Groupe Hospitalier Saint-Louis, Lariboisière, Fernand-Widal, CeGIDD, AP-HP, Paris, France; 31. Dermatology Department, Paris-Cité University, INSERM 976, Saint Louis Hospital, Paris, France; 32. Dermatology Department, Cochin Hospital, INSERM U1016, AP-HP, Paris, France; 33. Department of Pathology, Necker Hospital for Sick Children, AP-HP, Paris-Cité University, Paris, France; 34. Papillomavirus National Reference Center, Besançon Hospital, Besançon, France; 35. Department of Dermatology and Cutaneous Biology, Sidney Kimmel Medical College, Thomas Jefferson University, Philadelphia, Pennsylvania, USA.; 36. Jefferson Institute of Molecular Medicine, Thomas Jefferson University, Philadelphia, Pennsylvania, USA.; 37. Department of Pathology and Laboratory Medicine, UCLA Clinical Genomics Center, David Geffen School of Medicine at UCLA, Los Angeles, California, USA.; 38. Center for Applied Genomics, Children’s Hospital of Philadelphia, Philadelphia, Pennsylvania, USA; 39. Department of Pediatrics, Children’s Medical Center, Tehran University of Medical Sciences, Tehran, Iran; 40. Department of Neurology, University Hospitals Leuven, Leuven, Belgium; 41. Laboratory for Muscle Diseases and Neuropathies, Department of Neurosciences, KU Leuven, and Leuven Brain Institute (LBI), Leuven, Belgium; 42. KU Leuven Department of Microbiology, Immunology and Transplantation, Allergy and Clinical Immunology Research Group, KU Leuven, Leuven, Belgium; 43. Labtech Single-Cell@Imagine, Imagine Institute, INSERM UMR 1163, F-75015 Paris, France; 44. Pathogen Discovery Laboratory, Institut Pasteur, Paris Cité University, Paris, France.; 45. Paris Cité University, URP 7328 FETUS, Paris, France; 46. Microbiology Department, AP-HP, Necker Hospital for Sick Children, Paris, France; 47. Clermont Auvergne University, INSERM U1071, M2iSH, USC INRAE 1382; CHU Clermont-Ferrand, 3IHP, Department of Parasitology-Mycology, Clermont-Ferrand, France; 48. National Reference Center for Cryptosporidiosis, Microsporidia and Other Digestive Protozoa, Clermont-Ferrand, France; 49. Study Center for Primary Immunodeficiencies, Necker Hospital for Sick Children, AP-HP, Paris, France; 50. Centre for Molecular Medicine, Department of Medicine, Karolinska Institutet, Stockholm, Sweden; 51. College of Health and Life Sciences, Hamad Bin Khalifa University, Doha, Qatar; 52. Department of Human Genetics, McGill University, Montreal, Quebec, Canada; 53. Infections and Cancer Epidemiology, Infection, Inflammation and Cancer Program, German Cancer Research Center (DKFZ), Heidelberg, Germany; 54. Laboratory of Inborn Errors of Immunity, Department of Microbiology, Immunology and Transplantation, KU Leuven, Leuven, Belgium; 55. Department of Pediatrics, Leuven University Hospitals, Leuven, Belgium; 56. Specialized Immunology Laboratory of Dr. Shahrooei, Tehran, Iran; 57. Department of Laboratory Medicine, University Hospitals Leuven, Leuven, Belgium; 58. Department of Pediatrics, Necker Hospital for Sick Children, AP-HP, Paris, France; 59. Howard Hughes Medical Institute, New York, NY

**Keywords:** FLT3LG, FLT3L, FLT3, hematopoiesis, Human, dendritic cells, B cells, NK cells, Papillomavirus, primary immunodeficiency

## Abstract

FMS-related tyrosine kinase 3 ligand (FLT3L), encoded by *FLT3LG*, is a hematopoietic factor essential for the development of natural killer (NK), B cells, and dendritic cells (DCs) in mice. We describe three humans homozygous for a loss-of-function *FLT3LG* variant, with a history of various recurrent infections, including severe cutaneous warts. The patients’ bone marrow was hypoplastic, with low levels of hematopoietic progenitors, particularly myeloid and B-cell precursors. Counts of B cells, monocytes, and DCs were low in the patients’ blood, whereas the other blood subsets, including NK cells, were affected only moderately, if at all. The patients had normal counts of Langerhans cells and dermal macrophages in the skin but lacked dermal DCs. Thus, FLT3L is required for B-cell and DC development in mice and humans. However, unlike its murine counterpart, human FLT3L is required for the development of monocytes but not NK cells.

## In brief

Adult humans with inherited, complete FLT3L deficiency enable valuable insights into the role of the hematopoietic growth factor FLT3L in human hematopoiesis, revealing that FLT3L deficiencies impair monocyte, dendritic, and B-cell development but not NK cell development. This study reveals key differences in the role of FLT3L in humans versus mice.

## Graphical Abstract

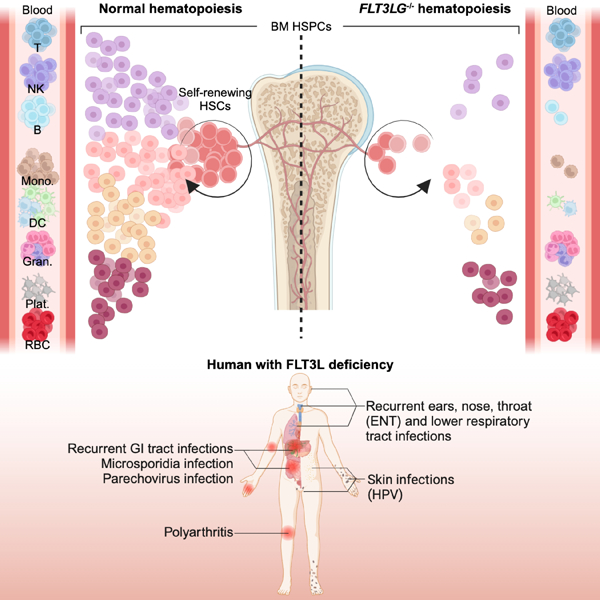

## Introduction

Hematopoiesis is the process of blood-cell formation from self-renewing hematopoietic stem cells (HSCs)^1^. HSC differentiation is driven by molecular cues leading to a gradual commitment to specific lineages and subsets through phenotypically well-defined stages. Recent advances suggest that lineage commitment occurs along an increasingly stable unipotent trajectory, with a limited amount of lateral transition during the successive progenitor steps^2,3^. Inborn errors of human hematopoiesis can underlie a broad spectrum of non-exclusive phenotypes, ranging from various cell deficits, including anemia, thrombocytopenia, and leukopenia, to various hematopoietic malignancies, including myeloid and lymphoid leukemias. Mutations impairing the fitness of HSCs and early progenitors result in a broad impairment of hematopoiesis, as exemplified by reticular dysgenesis, which was first reported in 1959^4,5^. Other inborn errors selectively block later stages of hematopoiesis, such as the development of granulocytes (severe congenital neutropenia) and B cells (agammaglobulinemia), two conditions widely considered to be the first two inborn errors of immunity to be described, in 1950^6–8^ and 1952, respectively^9,10^.

The hematopoietic growth factor Fms-like tyrosine kinase 3 ligand (FLT3L), encoded by *FLT3LG*, operates via its receptor FLT3^11^. Both molecules were identified in mice and humans in the early 1990s^12–17^. FLT3 is expressed on the surface of early hematopoietic precursors. FLT3L is broadly expressed, with levels highest in stromal bone marrow cells and T lymphocytes^17,18^. FLT3L is a transmembrane molecule that can act as a soluble cytokine after proteolytic release of its extracellular domain^16,19,20^. Soluble FLT3L binds FLT3 to form signal-transducing homodimers inducing the proliferation of early hematopoietic precursors^21^. In mice, *Flt3lg* knockout is associated with ~40% lower leukocyte counts in the blood and the spleen, ~80% lower splenic natural killer (NK) cell counts, ~70% lower splenic B-cell counts, and an almost complete absence of cDCs and pDCs in lymphoid and non-lymphoid tissues^22–25^. To date, the essential and redundant roles of human FLT3L remain unclear, because humans with genetic defects resulting in a deficiency of the growth factor or its receptor have never been described.

## Results

### Three siblings with viral, bacterial, and fungal infections

**Figure 1.**
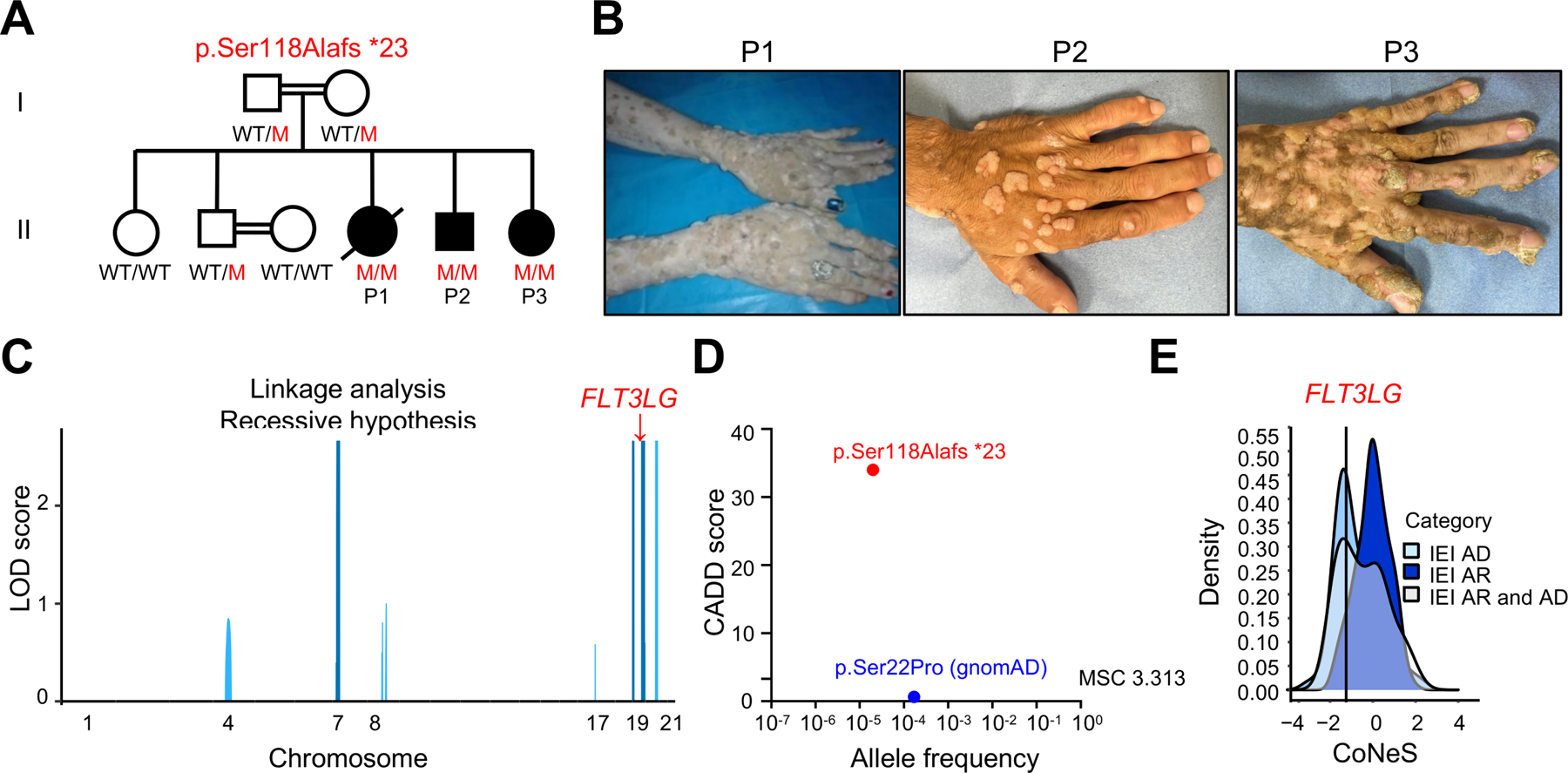
Three patients homozygous for a frameshift FLT3LG variant (A) Pedigree showing the familial segregation of the c.343delC (p. Ser118Alafs*23) *FLT3LG* allele. Solid black symbols indicate patients with FLT3L deficiency. Symbols linked with a double line indicate consanguinity. M: mutant, WT: wild type. (B) Cutaneous warts on the hands of P1, P2, and P3. (C) Genome-wide linkage analysis on gDNA from 7 family members, assuming AR inheritance with complete penetrance. All 6 linked regions with a high LOD score are shown in blue and positioned on chromosomes 7, 19, and 20; one of the regions on chromosome 19 included *FLT3LG* (red arrow). (D) Allele frequency and CADD score for the only *FLT3LG* variant reported in the homozygous state in public databases (showed in blue). The c.343delC variant is indicated in red. The dotted line corresponds to the mutation significance cutoff (MSC) for *FLT3LG*. (E) CoNeS score of *FLT3LG* is consistent with an AR trait.^33^ See also Figures S1 and S2.

We studied three siblings (P1, P2, P3) born in 1989, 1991, and 1995, respectively, to consanguineous Iranian parents (Figure 1A, Table S1). From the age of four years, they suffered from recurrent episodes of viral, bacterial, and fungal infections. The bacterial infections included pneumonia, otitis media, pharyngitis, and cellulitis, leading to conductive hearing loss with confirmed bilateral otosclerosis in P2 and P3. The patients presented viral infections, including chronic oral HSV infection, high levels of asymptomatic EBV viremia (P2, P3), high levels of human *Parechovirus* 1 (HPEV-1, *Parechovirus A*) replication in blood (P2) and stools (P2, P3), and HPV-driven skin lesions, including recalcitrant and disseminated HPV2^+^ common warts (P1-P3), HPV5^+^ epidermodysplasia verruciformis (P3) and HPV7^+^ genital warts (P3) (Figure 1B, S1A). In 2022, P2 had mild COVID-19 after two injections of the Sinopharm whole inactivated vaccine. All patients had recurrent diarrhea from early infancy and failure to thrive. However, P2 and P3 excreted high loads of microsporidia (*Enterocytozoon bieneusi*) and displayed signs suggestive of chronic cholangitis on ultrasound (i.e, biliary tract dilation) at the ages of 27 and 23 years, respectively. P1 and P3 developed seronegative polyarthritis at the age of 10 years. None of the three patients had any allergies or cancers. P1 died following a car accident at the age of 29 years. P2 and P3 are alive and are currently 31 and 27 years old, respectively. None of these patients suffered an adverse reaction after vaccination with several live attenuated vaccines in accordance with the national immunization program in Iran (i.e., following vaccination with bacillus Calmette-Guérin (BCG), oral polio, and measles, mumps, and rubella (MMR) vaccines). The three patients, thus, displayed broad susceptibility to infection, with HPV in particular.

### A homozygous frameshift variant of *FLT3LG*

We performed whole-exome sequencing (WES) for the entire family (the three patients, both parents, and two healthy siblings aged 38 and 35 years). The high percent homozygosity of P1 (5.5%), P2 (3.2%), and P3 (5.8%) on WES confirmed parental consanguinity. We performed a linkage analysis on WES data for the entire kindred, testing the hypothesis of an autosomal recessive (AR) trait with complete penetrance. Significant evidence of linkage was obtained for six regions (LOD (logarithm of the odds) score >2.5) (Figure 1C). Within the linked regions, non-synonymous variants that were both biallelic and rare (minor allele frequency (MAF)<0.01; Figure S1B and S1C) were detected for only seven protein-coding genes. Only one of these variants was predicted to be loss-of-function (pLOF), and this variant had never been reported in the homozygous state in any public database. This variant, confirmed by targeted Sanger sequencing (Figure S1D), was a single-nucleotide homozygous frameshift deletion leading to a premature stop codon (p.Ser118Alafs*23) in the *FLT3LG* gene (c.343delC). *In silico* analysis predicted the variant to be damaging, with a combined annotation-dependent depletion (CADD) score of 34, well above the mutation significance cutoff (MSC) of 3.13 for this gene (Figure 1D)^26,27^. The variant was reported in the heterozygous state, with an allele frequency of 0.00001987 in gnomAD V3.1.2^28^, and 0.00015 in our inhouse Iranian cohort of more than 6000 individuals. Neither this nor any other pLOF variant was reported in the homozygous state in public databases^28–32^. There is only one missense *FLT3LG* variant present in the homozygous state in public databases (p.Ser22Pro), with an allele frequency of 0.00005920 in gnomAD V3.1.2 (Figure 1D). Accordingly, the CoNeS score of *FLT3LG* is consistent with a gene under strong negative selection (Figure 1E)^33^. Moreover, we showed that *Flt3lg*^−/−^ mice were vulnerable to the murine papillomavirus MmuPV1 (Figure S2). These findings suggested that the homozygous c.343delC (p.Ser118Alafs*23) variant of *FLT3LG* might underlie the clinical phenotypes of these three patients.

### Human *FLT3LG* encodes at least seven transcripts

The *FLT3LG* mRNA is detectable in most tissues^34–37^ but its levels are highest in T cells. Alternative splicing events are frequent for the *FLT3LG* mRNA, as attested by RNA sequencing (RNA-seq) data obtained with human naïve CD8^+^ T cells (Figure S3A), but the relative abundance and function of the alternative full-length mRNA transcripts have been studied in less detail in human cells than in mice^35,38,39^. The patients’ variant is present in only some of these alternative splicing variants. We therefore studied the expression of *FLT3LG* transcripts in T-cell blasts from healthy donors (Figure S3B). We found that there were at least seven alternative transcripts (T1-T7), with frequencies between 4% and 34% of total *FLT3LG* mRNA. Together, the canonical T1/T2 transcripts accounted for only ≃34% of the total *FLT3LG* transcripts in control T-cell blasts (Figure S3B and S3C). The first four coding exons (exons 2 to 5), which encode the first 114 amino acids of the canonical functional protein encoded by T1/T2, are conserved in all seven transcripts. The biological activity of FLT3L is conferred by the extracellular domain^17^, so some non-canonical transcripts (T3-T7) may be expressed and functional.

### Two soluble isoforms of FLT3L are released by cleavage or secretion

We investigated whether the seven *FLT3LG* transcripts were expressed in a HEK293T overexpression system. RT-qPCR revealed that all seven transcripts were expressed at similar levels (Figure S3D). By flow cytometry, we observed strong cell-surface expression of the canonical protein encoded by T1/T2, weak cell-surface expression of the proteins encoded by T3, T4 and T5, and no detectable cell-surface expression of the proteins encoded by T6 and T7 (Figure S3E). Western blotting on total cell extracts detected high levels of a protein with a molecular weight (MW) higher than predicted from the amino-acid sequences of T1/T2 and T7, probably due to posttranslational modifications (Figure S3F). T3, T4, and T5 protein levels were lower than those for T1/T2 and T7 (Figure S3F). No protein was detected for T6 (Figure S3F). Furthermore, immunoblotting with an N-terminal antibody or enzyme-linked immunosorbent assay (ELISA) revealed that only T1/T2 and T7 gave rise to detectable amounts of protein in the supernatant of HEK293T cells 48 hours post transfection (Figure S3F and S3G). Release of the extracellular domain by enzymatic cleavage^19^ resulted in the truncated canonical protein encoded by T1/T2 having a lower MW in the supernatant than in the whole-cell extract (Figure S3F). This cleaved protein could not be detected with the anti-V5 antibody (against a C-terminal tag), whereas the T7 protein, which was secreted due to the lack of the cleavage site and the transmembrane domain (skipping of exon 7), was detectable (Figure S3F). Thus, these data show that human *FLT3LG* encodes two main isoforms. The first (the canonical protein encoded by T1 or T2) is expressed at the cell membrane and can be released into the supernatant by enzymatic cleavage. The second (encoded by T7) is secreted due to the absence of a transmembrane domain.

### Only one transmembrane FLT3L and the secreted FLT3L are functional

**Figure 2.**
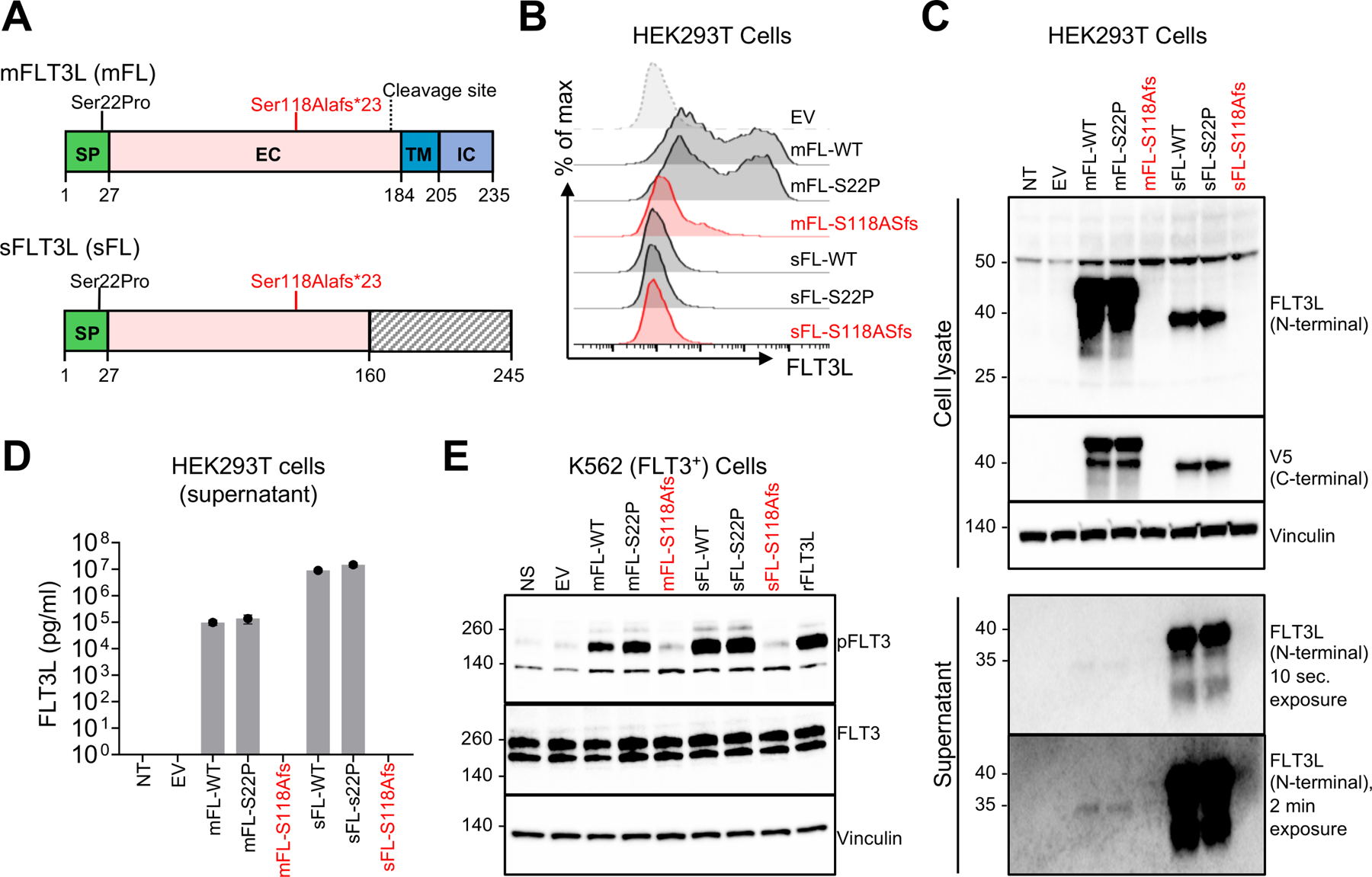
The patients’ FLT3LG mutation is loss-of-expression and loss-of-function in vitro. (A) Schematic representation of the two functional FLT3L isoforms. The different domains are indicated. The position of the p.Ser118Alafs*23 mutation is indicated in red and that of the p.Ser22Pro mutation is indicated in black. mFLT3L (mFL): membrane-bound FLT3L. sFLT3L (sFL): secreted FLT3L. SP: signal peptide. EC: extracellular domain. TM: transmembrane domain. IC: intracellular domain. (B) Surface FLT3L expression in an overexpression system, as assessed by FACS on HEK293T cells 48 hours after transfection with an empty vector (EV), or a vector encoding the WT sFL or mFL isoform, or the indicated *FLT3LG* variants. (C) WB analysis of cell-culture supernatants or cell lysates obtained from HEK293T cells, as in B. (D) Soluble FLT3L protein determination by ELISA on the cell-culture supernatant of HEK293T cells, as in B and C. Bars indicate the mean and SD. (E) Phospho-FLT3 (pFLT3) levels in K562 cells transduced with FLT3, as assessed by WB, after 5 minutes stimulation with the supernatant collected from HEK293T cells 48 h after transfection. rhFLT3L was used as a positive control. All the data shown are representative of three independent experiments. See also Figures S3A-J.

As a protein was detected for five isoforms (encoded by T1/T2, T3, T4, T5 and T7), we investigated the functional activity of these proteins *in vitro*. K562 cells stably transduced with FLT3, the only known receptor of FLT3L, were stimulated with the supernatant of HEK293T cells transfected with an empty vector or a construct containing one of the six coding sequences (Figure S3H). With this system, consistent with the findings for the supernatant of transfected HEK293T cells, the supernatants of T1/T2 and T7 induced FLT3 phosphorylation to levels similar to those achieved with a commercial human recombinant FLT3L used as a positive control. As expected, no FLT3 phosphorylation was detected after the stimulation of K562 cells with the supernatant of HEK293T cells transfected with T3, T4, T5, or T6 (Figure S3H). In addition, despite weak expression at the cell surface (Figure S3E), cells transfected with T3, T4, or T5 were unable to induce FLT3 phosphorylation in K562 cells when the two cell lines were incubated together to allow contact between cells (Figure S3I). Thus, only the isoforms encoded by T1/T2 and T7 were functional, both of which contain amino-acid residues that are affected in the patients. We named the canonical surface-expressed FLT3L encoded by T1 or T2 mFLT3L (mFL), and the secreted FLT3L growth factor encoded by T7 sFLT3L (sFL) (Figure 2A).

### The p.Ser118Alafs*23 variant is LOF *in vitro*

We assessed the impact of the p.Ser118Alafs*23 variant found in the patients on FLT3L function *in vitro*. We transfected HEK293T cells with an empty vector or a vector encoding mFL or sFL, with the WT, p.Ser118Alafs*23 (S118Afs) or p.Ser22Pro (S22P) variant. In this system, RT-qPCR revealed that mRNA levels were similar for all constructs (Figure S3J). However, unlike the WT mFL or sFL, the p.Ser118Alafs*23 variant resulted in an absence of both protein isoforms in flow cytometry experiments and western blots on total cell extracts or cell-culture supernatants (Figure 2B and 2C). A re-initiation of translation downstream from the premature stop codons was excluded by immunoblotting total cell extracts or cell-culture supernatant with a C-terminal antibody against the V5 tag (Figure 2C). The absence of p.Ser118Alafs*23 protein shedding or secretion into the supernatant was confirmed by ELISA (Figure 2D). Furthermore, the supernatant of HEK293T cells transfected with the mutant mFL or sFL was unable to induce FLT3 phosphorylation in the K562 reporter assay (Figure 2E). As expected, both the membrane and secreted isoforms carrying the p.Ser22Pro variant (the only coding variant found in the homozygous state in public databases) were produced in normal amounts and functional (Figure 2B-E), suggesting that autosomal recessive FLT3L deficiency is exceedingly rare globally. Together, these data confirm that the p.Ser118Alafs*23 variant is LOF *in vitro,* due to an impairment of the expression of both mFL and sFL.

### Abolition of endogenous FLT3L production

**Figure 3.**
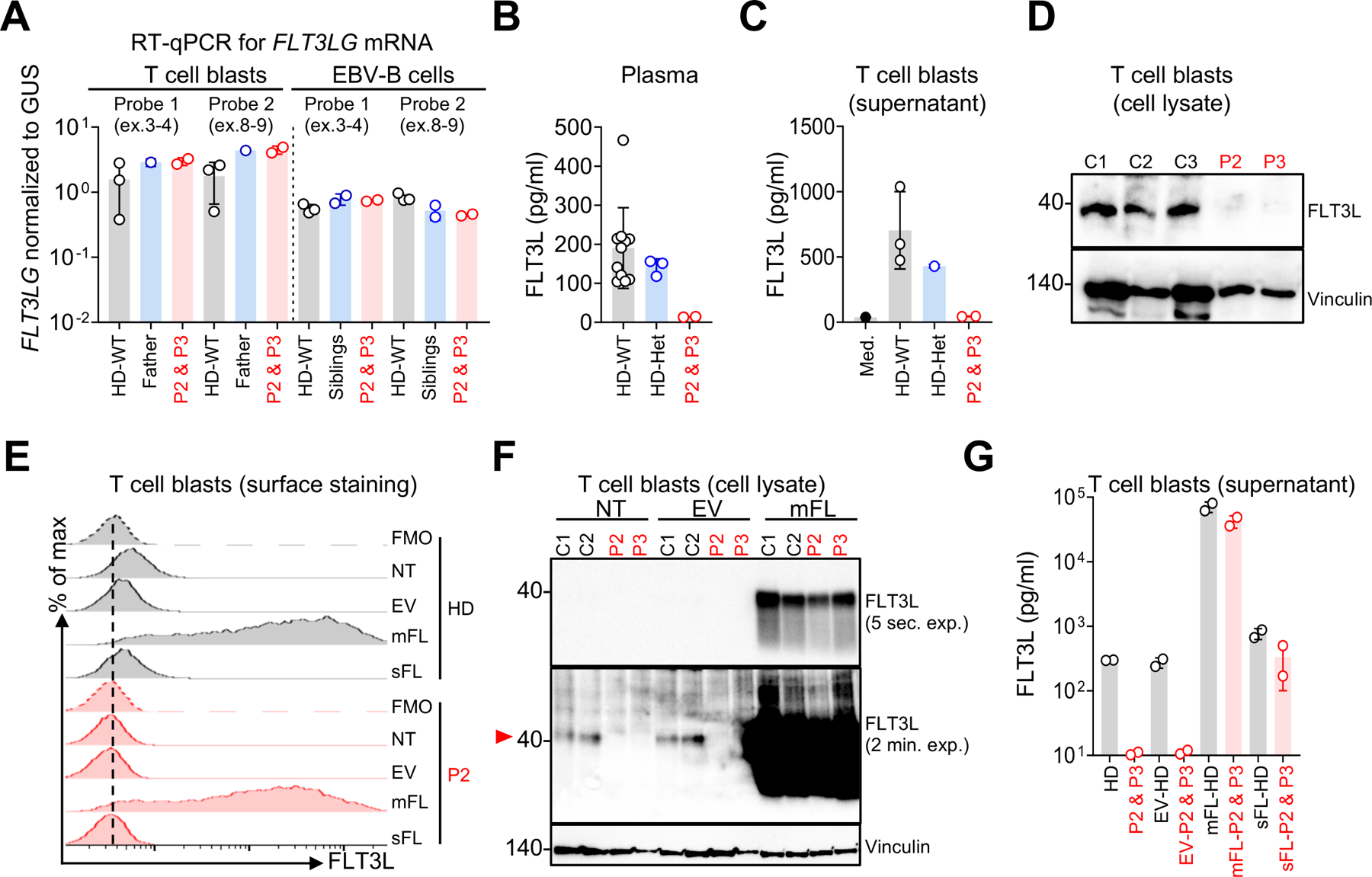
Biological significance of the FLT3LG allele and rescue of the phenotype (A) Total mRNA was extracted from the T-cell blasts of three HDs, a heterozygous control (the father), P2, and P3, and EBV-B cells from three HDs, both healthy siblings, P2, and P3. Total mRNA was subjected to RT-qPCR for the assessment of *FLT3LG* expression with two different probes. Data are displayed as 2^−ΔCt^ values normalized against the expression of endogenous GUS. Bars represent the mean values and SD. (B) Soluble FLT3L determination by ELISA in plasma samples from 12 HDs (all HDs were adults; male or female, and from multiple ethnic groups), the three heterozygous family members, P2, and P3. Bars indicate the mean and SD. (C) Soluble FLT3L determination by ELISA on supernatant samples collected from T-cell blasts from three adult HDs, the heterozygous father, P2, and P3. The medium (Med) from the T-cell culture was used as a negative control. Bars represent the mean and SD. (D) WB analysis of cell lysates obtained from T-cell blasts from three unrelated HDs, P2 and P3. Vinculin was used as a loading control. (E-G) T-cell blasts from patients and controls were either left non-transduced (NT) or were transduced with lentiviruses generated with an empty vector (EV) or with vectors containing the WT sFL or mFL cDNA. (E) Cell-surface FLT3L expression assessed by flow cytometry with a monoclonal Ab (EP1140Y). (F) WB analysis of FLT3L on T-cell blasts from P2, P3, and two HDs. Vinculin was used as a loading control. The red arrow shows the band corresponding to the endogenous FLT3L. (G) Soluble FLT3L protein determination by ELISA on the cell-culture supernatant of T-cell blasts from P2, P3, and two HDs. Bars represent mean with SD. See also Figure S3K.

We tested the impact of the p.Ser118Alafs*23 genotype on the expression of endogenous *FLT3LG* in the patients’ plasma and cells. FLT3L expression in leukocytes is strongest in T lymphocytes^39^. We analyzed FLT3L expression in T-cell blasts and EBV-transformed B cells. RT-qPCR showed that the p.Ser118Alafs*23 mutation did not affect endogenous *FLT3LG* mRNA levels (Figure 3A). Accordingly, in our cDNA cloning experiment with T-cell blasts (Figure S3B), the distribution of transcripts (T1-T7) was similar in the patients and controls. However, FLT3L was undetectable by ELISA in the patients’ T-cell blast culture supernatants and plasma, whereas it was detected in the controls and the heterozygous family members, in whom FLT3L levels were similar (Figure 3B and 3C). Western blots of whole-cell lysate and flow cytometry for cell-surface expression detected low levels of FLT3L in control T-cell blasts but not in T-cell blasts from the patients (Figure 3D and 3E). We then stably transduced T-cell blasts from P2 and P3 with a lentiviral vector encoding the WT mFL and sFL (Figure 3E-G and S3K). As expected, only the WT mFL construct restored FLT3L expression at the cell surface of the patients’ T-cell blasts, as demonstrated by flow cytometry (Figure 3E). Western blots and ELISA showed that the WT mFL isoform restored FLT3L expression in cell lysates and cell-culture supernatants (Figure 3F and 3G). The WT sFL isoform restored protein detection by ELISA in the supernatant, but not by western blotting on total cell lysates (Figure 3F and 3G), possibly due to immediate secretion of the protein. Endogenous FLT3L in plasma or the supernatant of T-cell blasts from controls was unable to induce detectable FLT3 phosphorylation in our K562 reporter assay (not shown), and could not be used to assess FLT3L activity at more physiological levels. Thus, homozygosity for the p.Ser118Alafs*23 variant underlies complete FLT3L deficiency in these patients. FLT3L expression can be restored by transducing the patients’ cells with the WT mFL or sFL cDNA.

### FLT3L deficiency is associated with hypoplastic bone marrow

**Figure 4.**
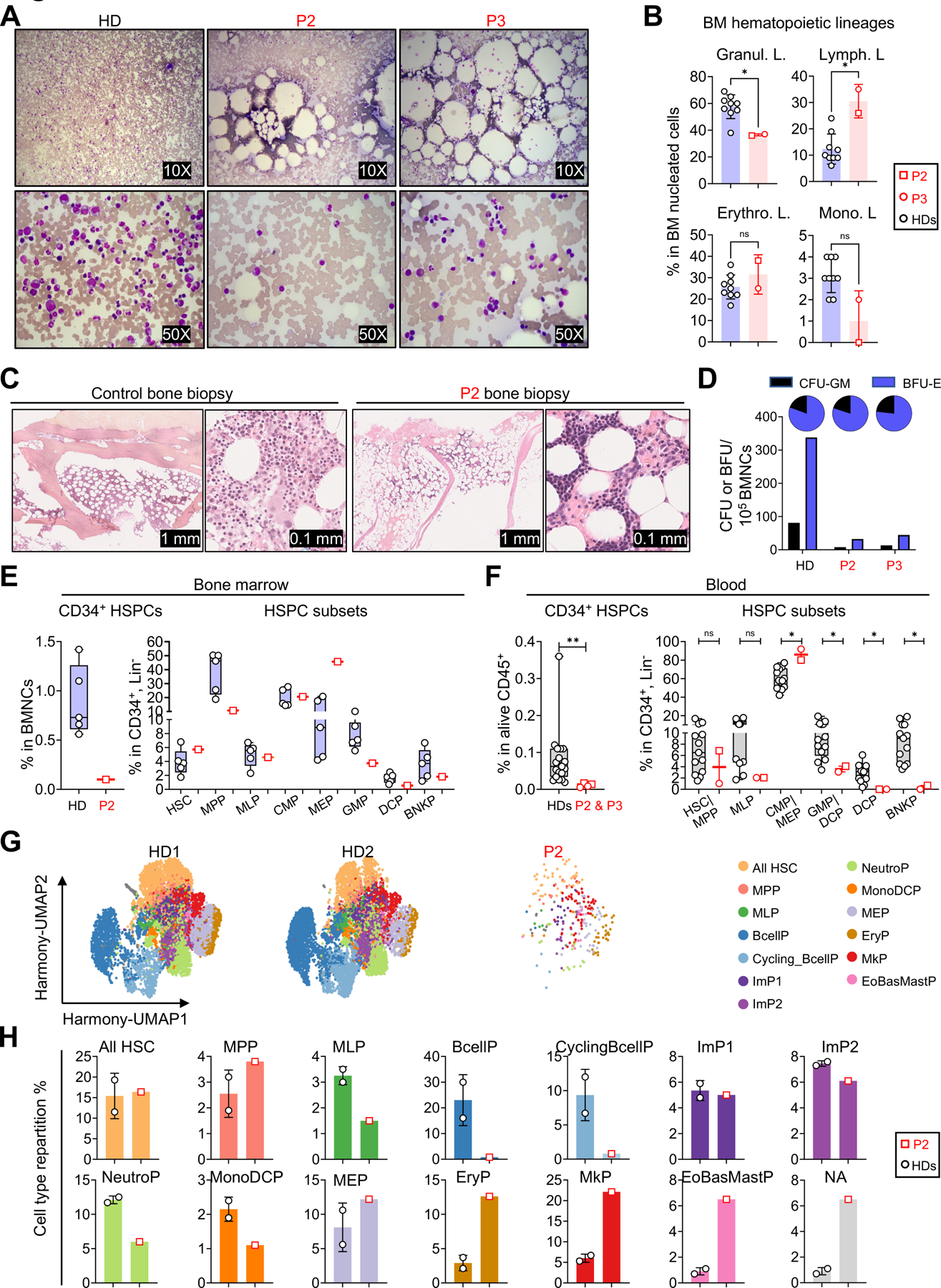
Bone-marrow and hematopoietic progenitors in FLT3L-deficient patients (A) Light microscopy images of bone marrow smears, for P2, P3 and one age-matched adult HD, after May-Grumwald-Giemsa (MGG) staining. The nucleated cells appear violet. The round, unstained areas correspond to adipocytes, dissolved by MGG staining. (B) Scatter dot plots visualizing the proportions of the indicated hematopoietic lineages among total nucleated cells from P2, P3, and HDs. Bars represent the mean and SD. Two-tailed Mann–Whitney *U* tests were performed to assess the difference between the patients and nine HDs (4 males and 5 females, aged 23–43, all from European ancestries). *P-value≤0.05, ns: not significant. (C) Bone biopsy for P2 and an age-matched control, showing hypocellularity in P2. At higher magnification of P2’s slide, we observed mostly mature erythroblasts and megakaryocytes, whereas the granulocytic lineage appeared largely hypoplastic. (D) Counts of hematopoietic progenitors (CFU & BFU) in a colony-forming assay performed 13 days after culture with cells from P2, P3, and an adult HD. The pie charts display the proportions of the total colonies corresponding to CFU-GM and BFU-E. (E) Left: frequency of CD34^+^ hematopoietic stem and progenitor cells (HSPCs) from the BM samples of P2 and 5 adult HDs (all males, aged 24–43). Right: frequency of hematopoietic stem cells (HSCs), multipotent progenitors (MPPs), multilymphoid progenitors (MLPs), common myeloid progenitors (CMPs), megakaryocyte–erythroid progenitors (MEPs), granulocyte–monocyte progenitors (GMPs), dendritic cell progenitors (DCPs), and B cell–NK progenitors (BNKPs) in CD34^+^Lin^−^ cells from P2 and 5 HDs. Data are presented as individual values (min to max). Boxplots show the median and interquartile range (IQR). (F) Left: frequency of CD34^+^ cells from the PBMCs of P2, P3, and 19 HDs. Right: frequency of HSCs/MPPs, MLPs, CMPs/MEPs, GMPs/DCPs, DCPs, and BNKPs in P2, P3 and 13 HDs (all HDs were adults, male or female, and from multiple ethnic groups). Data are presented as individual values (min to max). Boxplots show the median and IQR. Two-tailed Mann–Whitney *U* tests were performed to assess the difference between the patients and the controls. *P-value≤0.05, **P-value≤0.01, and ns: not significant. The value for a single time point was used for each individual in all statistical analyses; however, the graphs for the patients display different values measured at different time points. (G-H) Single-cell RNA sequencing on CD34^+^ cell-enriched HSPCs from the bone marrow of P2 and two HDs (one male, one female, aged 44 and 53, respectively). (G) Unsupervised analysis of CD34^+^ HSPCs from HDs (HD1: 7604 cells and HD2: 7298 cells) and P2 (262 cells), represented as two-dimensional Uniform manifold approximation and projection (UMAP) plots. All HSCs: HSC and HSC-enriched, MPP, MLP, ImP1 & ImP2: immature myeloid progenitors, NeutroP: neutrophil progenitors, MonoDCP: monocyte and dendritic cell progenitors, PreB: B cell progenitors, MEP: megakaryocyte and erythrocyte progenitors, EryP: erythroid progenitors, MkP: megakaryocyte progenitors, EoBasMastP: eosinophil, basophil and mast cell progenitors, NA: not annotated. (H) Proportion of each HSPC subpopulation among the CD34^+^ HSPC shown in 5G. Bars represent the mean and SD. See also Figure S4.

*FLT3LG*-knockout mice have low levels of hematopoietic progenitors, especially DC progenitors and B-cell precursors, in their bone marrow (BM) (Table S1)^24,40^. We first studied BM smears from P2 and P3 to characterize the impact of FLT3L deficiency on cellularity and progenitor populations. BM cellularity was low in both patients, with lipid structures observed in the smears (Figure 4A). A myelogram revealed general medullary hypoplasia, of the myeloid lineage in particular, resulting in an increase in the proportions of lymphocytes and late erythroblasts (acidophilic erythroblasts) (Table S1). The proportion of cells of the granulocyte lineage among the nucleated cells detected in the BM myelogram was about 40% lower, in both patients, than the mean value for eight unrelated aged-matched healthy donors, whereas the proportion of lymphocytes was more than 50% higher (Figure 4B). A biopsy from P2 showed that his bone marrow was hypocellular (ratio hematopoietic cells/adipocytes, (10 to 20%) versus 60% for an age-matched control individual). It was also characterized by a granulocytic lineage that was highly hypoplastic relative to the erythroblastic or megakaryocytic lineages (Figure 4C, and S4A). A diagnosis of bone marrow failure was therefore established. In addition, the BM mononuclear cells (BMNCs) of the patients had a low capacity for generating myeloid and erythroid progenitors, as shown by an ~8-fold decrease in the number of colony-forming units (CFU-GM) and burst-forming units (BFU-E) in standard differentiation assays (Figure 4D). Together, these data show that the bone marrow of the FLT3L-deficient patients was severely hypoplastic, probably due to an impairment of HSPC differentiation or maintenance.

### FLT3L deficiency results in a major decrease in HSPCs

Consistent with the findings reported above, standard flow cytometry showed that the percentage of CD34^+^Lin^−^ HSPCs among BMNCs was much lower in P2 than in controls (~9-fold lower than the mean value for HDs) (Figure 4E, left). We then obtained an enriched population in residual CD34^+^ HSPCs from P2’s bone marrow, on which we performed extensive phenotyping to characterize its CD34^+^ HSC and progenitor content (Figure 4E, S4B, and S4C). We found that the proportions of multipotent progenitors (MPPs), granulocyte/monocyte progenitors (GMPs), and dendritic cell progenitors (DCP) were slightly lower than those in healthy donors, whereas those of megakaryocyte-erythroid progenitors (MEPs) were higher in P2 than in controls (Figure 4E and S4C). The frequencies of common myeloid progenitors (CMPs), multipotent lymphoid progenitors (MLPs) and B and NK progenitors (BNKPs) were within the normal range (Figure 4E and S4C). We then used a strategy described elsewhere^41^ to perform a complementary analysis of the frequencies of circulating hematopoietic precursors in P2 and P3 based on an analysis of the CD34^+^Lin^−^ population in peripheral blood mononuclear cells (PBMCs) (Figure 4F and S4D). As in the bone marrow, the frequency of CD34^+^ HSPCs among PBMCs was lower in the patients than in 13 unrelated controls (~9-fold lower) (Figure 4F). The frequencies of HSC/MPP and MLPs among the rare HSPCs were in the normal range (P2) or were lower (P3) than those in controls. The frequencies of the CMP/MEP progenitor subsets were high in both patients. GMP levels were low and DCPs were absent in both patients. BNKPs were undetectable in both patients.

### The residual HSPCs of FLT3L-deficient individuals are biased toward the megakaryocytic and erythrocytic lineages

We performed single-cell RNA sequencing on CD34^+^ HSPCs isolated from the BMNCs of P2 and two healthy controls matched for age and sex. The results were analyzed with a previously described clustering approach (Figure 4G and H)^42^. Due to the limited sample size, and the rarity of CD34^+^ cells in FLT3L-deficient patients, only a small number of HSPCs could be analyzed for P2. Nevertheless, this analysis confirmed the normal frequencies of HSCs and MPPs among P2’s HSPCs, and revealed a frequency bias toward the megakaryocytic-erythroid pathway, with a large proportion of immediate progenies of MEP cells, including MkPs (megakaryocyte-committed progenitors) and EryPs (erythroid progenitors). Consistent with our flow cytometry analysis, the frequencies of myeloid progenitors (NeutroPs and MonoDCPs, together corresponding to GMPs) were slightly lower in the patient than in the two controls. For lymphoid progenitors, MLP levels were slightly lower, and B-cell precursor (BcellPs and CyclingBcellPs) levels were considerably lower in the patient than in the two controls (Figure 4G and H). In a pseudobulk analysis, FLT3 mRNA levels were normal in P2’s HSCs (the most immature cells), whereas they were low in bulk HSPC analysis, suggesting that the development or survival of FLT3^+^ progenitors may be poor in FLT3L-deficient individuals (Figure S4E). Overall, our data show that the rare BMs and circulating HSPCs of the patients are enriched in megakaryocytic and erythroid progenitors, and that the frequencies of B-cell, monocyte, granulocyte, and DC progenitors are low.

### Monocytopenia in FLT3L-deficient patients

**Figure 5:**
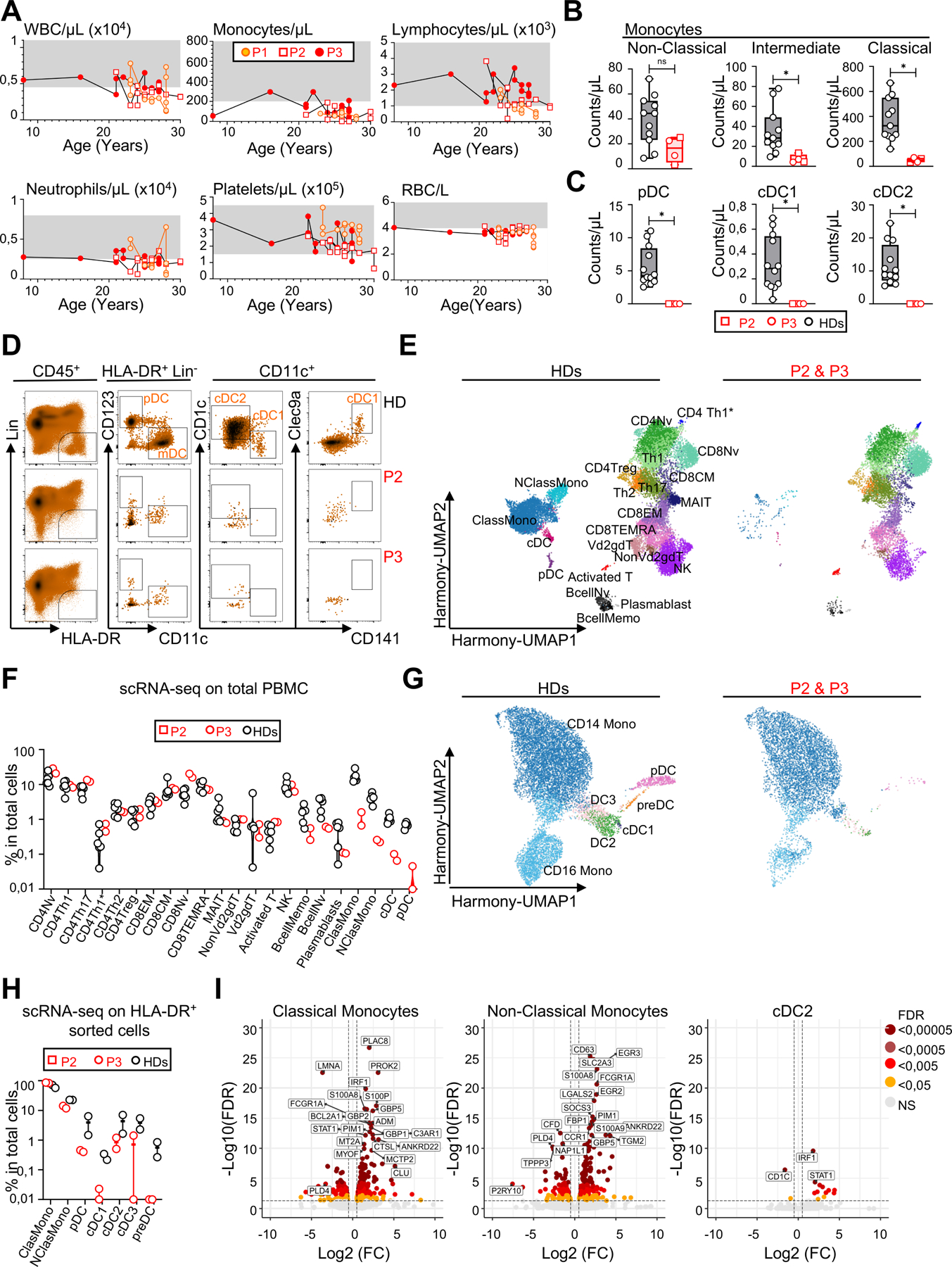
Hematological profile, including the myeloid- and lymphoid-related immune compartments in the peripheral blood of FLT3L-deficient patients (A) Absolute numbers for peripheral blood cell subsets in P1, P2, and P3 over time. The reference values for each parameter are indicated by shaded areas. WBC: white blood cells, RBC: red blood cells. (B) Absolute counts for peripheral blood monocyte subsets assessed by CyTOF in P2, P3, and 11 HDs (all HDs were adults, male or female, and from multiple ethnic groups). Data are presented as individual values (min to max). Boxplots show the median and IQR. Two-tailed Mann–Whitney *U* tests were performed to assess the difference between patients and controls. *P-value≤0.05, and ns: not significant. The value for a single time point for each individual was used for all statistical analyses. However, the graphs for the patients display different values measured at different time points. (C) Absolute counts of peripheral blood dendritic cells assessed by CyTOF in P2, P3, and 12 HDs (all HDs were adults, male or female, and from multiple ethnic groups). Data presentation and statistics are as in B. (D) Flow-cytometry analysis of three subsets of circulating DCs assessed in P2, P3, and one HD after the acquisition of more than >10^7^ events, with more than 5 million live cells/sample. The first gating shows the HLA-DR^+^Lin^−^ (CD3, CD19, CD20, CD14, CD16, CD56) population gated on live CD45^+^ cells after the removal of doublets. (E) UMAP clustering for 5′ scRNA-seq performed on total PBMCs from P2, P3 and six HDs (all HDs were adults, male or female, and from multiple ethnic groups). The 22 clusters were identified on the basis of cell marker expression analysis. (F) Frequencies of the clusters shown in 5E. (G) UMAP clustering for 5′ scRNA-seq performed on the flow cytometry-sorted HLA-DR^+^Lin^−^ (CD3, CD19, CD20, CD56) populations from P2, P3 and two adult HDs (one male and one female, from the same ethnic group as the patients). The seven clusters targeted are identified on the basis of cell marker expression analysis^43^. (H) Frequencies of the clusters identified in 5G. (I) Volcano plot showing the results of a differential gene expression (DEG) analysis of the experiment in 5G and H, comparing normalized gene expression levels between patients and controls. Genes with a log_2_ fold change (log2FC) in expression and a FDR< 0.0005 were considered significant. A color code indicates the level of significance. See also Figure S5.

We then investigated the impact of complete FLT3L deficiency on complete blood counts in all three patients over a period of 5 (P1), 9 (P2) and 19 (P3) years of follow-up. Hemoglobin levels and red blood cell (RBC) counts were slightly low in all three patients and were considered to correspond to moderate normocytic anemia with normal reticulocyte counts (Figure 5A, S5A and Table S1). This moderate anemia was accompanied by low ferritin levels in P3, with a systematically high erythrocyte sedimentation rate (ESR), and hypergammaglobulinemia in all three patients (Figure S5A and Table S1). The observed anemia was therefore probably multifactorial, with iron deficiency in P3, and hemodilution due to hypergammaglobulinemia in all three patients. Total platelet counts and platelet morphology were normal, but had decreased at last follow-up for P2 and P3 (Figure 5A and Table S1). Total white blood cell (WBC) counts oscillated between low and normal values (Figure 5A and Table S1). All the patients consistently had profound monocytopenia, except for P3, who presented normal values on rare occasions (Figure 5A and Table S1). The levels of eosinophils and basophils were in the normal range (Table S1). Neutrophil counts were in the lower part of the control range or, occasionally, lower (Figure 5A, Table S1). Total lymphocyte counts were normal (Figure 5A and Table S1). Thus, patients had normal platelet and lymphocyte counts, near-normal neutrophil and red blood cell counts, and monocytopenia.

### FLT3L deficiency impairs the differentiation of monocytes and DCs

We performed a deep immunophenotyping by conventional cytometry on fresh PBMCs, and mass cytometry (cytometry by time of flight [CyTOF]) on fresh whole blood and BM (Figure 5B-D, S5B-E, and Table S1). Comparisons with healthy controls confirmed that the patients had profound monocytopenia in both the blood and BM, affecting all monocyte subsets, including classical (CD14^+^CD16^−^), intermediate (CD14^+^CD16^+^), and non-classical (CD14^dim^CD16^+^) monocytes (Figure 5B, S5B and S5E). Both patients tested (P2 and P3) had almost no CD123^+^ plasmacytoid DCs (pDCs) or conventional DC2 (cDC2) in the blood, and conventional DC1 (cDC1) were undetectable by CyTOF and flow cytometry on multiple occasions (Figure 5C, D, S5E and Table S1). Monocytopenia and the almost total absence of DCs were confirmed in a scRNA-seq experiment performed on total PBMCs (Figure 5E and F). We increased the resolution for myeloid antigen-presenting cells (APCs), by using FACS to sort HLA-DR^+^Lin^−^ cells from the PBMCs of P2, P3 and two controls, to achieve an enrichment in monocytes and DCs before scRNA-seq. Myeloid cell subsets were analyzed as previously described^43–45^. The frequencies of non-classical monocytes and of all DCs among sorted APCs were low, suggesting that, despite their very low counts, classical monocytes nevertheless constituted the major myeloid source of APCs in the patient. This approach did not detect DC precursors (preDCs) in either of the patients tested (Figure 5G and H). We detected rare pDCs and cDC2s in both patients, and cDC1s and cDC3s in P2 only, suggesting that the differentiation of these cells was severely impaired, but not completely abolished (Figure 5G and H). A comparison of the differential gene expression (DGE) profiles of patients and healthy controls revealed alterations in the monocytes and cDC2s of the patients (Figure 5I). *IRF1*, *STAT1*, *GBP5*, and *GBP2*, which encode proteins involved in interferon signaling pathways, were upregulated in the patients’ classical monocytes (Figure 5I). *IRF1* and *STAT1* were also upregulated in the patients’ cDC2 subset. In addition, genes involved in inflammatory responses, such as *S100A8* and *S100A9*, were upregulated in the classical monocytes and cDC2s of the patients (Figure 5I). No significant alterations were observed in the patients’ pDCs. Together, these data demonstrate a near total absence of DCs in the blood of the patients. Transcriptomic analysis of the residual monocytes and the rare cDC2s found in patients suggested an inflammatory state, possibly reflecting persistent infections.

### FLT3L deficiency has no effect on T-cell differentiation and function but impairs B-cell differentiation

**Figure 6.**
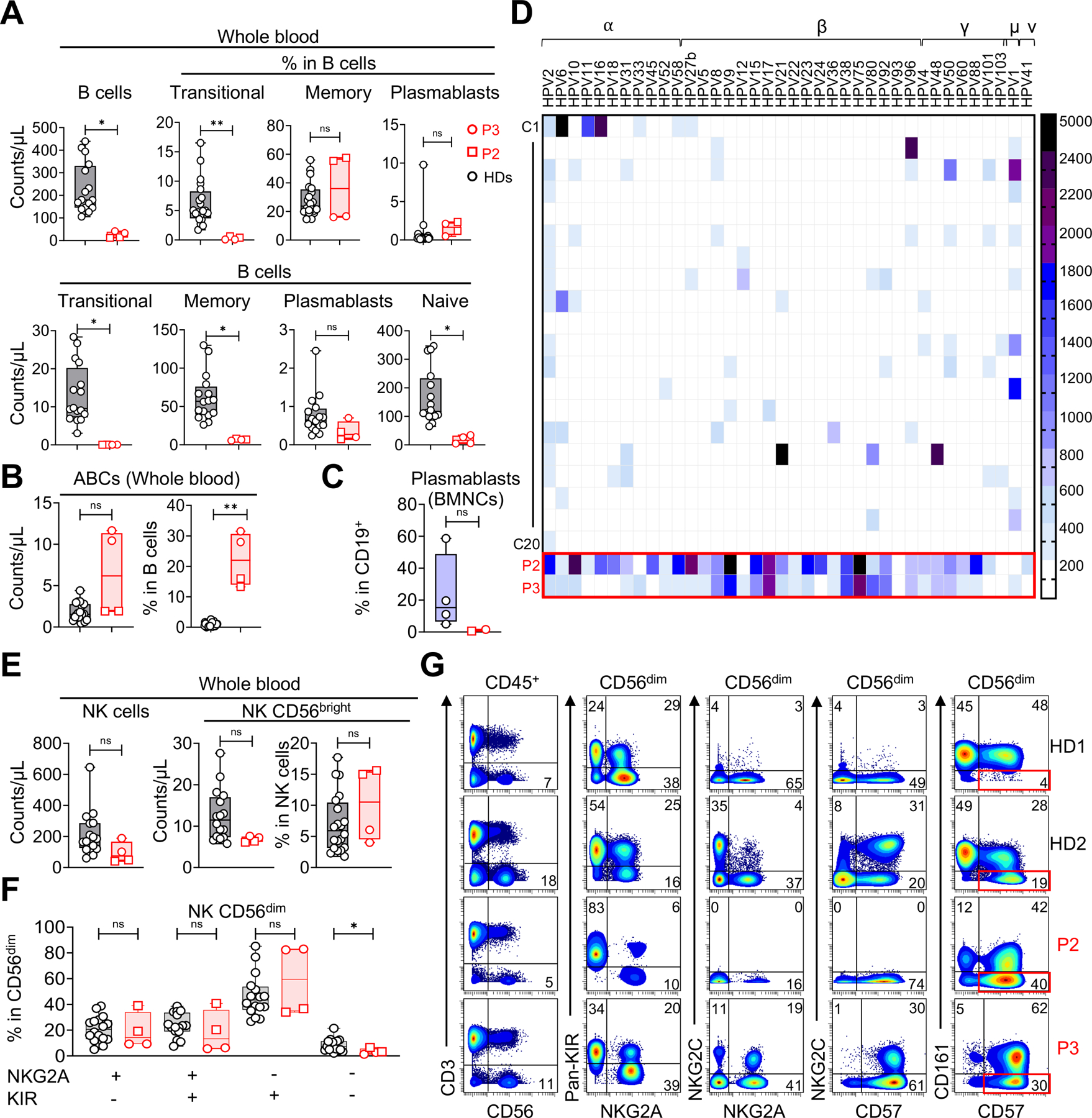
B-cell and NK cell features in FLT3L-deficient patients (A) Absolute blood counts of peripheral B-cell subsets, as defined by CyTOF, for P2, P3, and 20 HDs (all HDs were adults, male or female, and from multiple ethnic groups). Data are presented as individual values (min to max). Boxplots show the median and IQR. Two-tailed Mann–Whitney *U* tests were performed to assess the difference between the patients and the controls. *P-value≤0.05, **P< 0.01, ns: not significant. The value for a single time point for each individual was used for all statistical analyses. However, the graphs for the patients display different values measured at different time points. (B) Absolute blood counts and proportions among B cells of age-related B cells (ABCs), as defined by CyTOF, for P2, P3, and 20 HDs (as in A). Data presentation and statistics are as in A. (C) Proportions of plasmablasts among the CD19^+^ B cells of BMNCs, as defined by CyTOF, for the BM samples of patients and four adult HDs. Data presentation and statistics are as in A. (D) Heatmap showing the relative values for antibody reactivity against 38 different HPV types measured in a Luminex-based serological analysis performed on plasma samples from P2, P3 and 20 adult controls (male or female, multiple ethnic groups; each row represents a single serum sample). MFI values above 200 were considered positive. (E) Absolute counts of total NK cells and CD56^bright^ NK cells, and the proportion of CD56^bright^ cells among NK cells from P2, P3, and 16 HDs (all HDs were adults, male or female, and from multiple ethnic groups). Data presentation and statistics are as in A. (F) Percentages of CD56^dim^ NK cell differentiation subsets, as defined on the basis of pan-KIR and NKG2A expression detected by flow cytometry^50^, for P2, P3 and 16 HDs (as in E). Data presentation and statistics are as in A. (G) Flow-cytometry analysis showing that the CD56^dim^ NK cell subsets of the two patients include adaptive NK cells. This subset can be identified as NKG2C^+/−^CD161^−^CD57^+^ cells. Two HDs were included as examples. HD1 has no adaptive NK cells. HD2 has a large subset of adaptive NK cells. Note that P2 is homozygous for a frequent deletion in *KLRC2*, which encodes NKG2C^49^. See also Figures S5E, S6 and S7.

Despite a profound deficiency of multiple myeloid APCs, the patients’ T cells developed and differentiated *in vivo*, and responded to APC-independent stimuli *in vitro* and *ex vivo* (Fig. S5C and S5D, Fig. S6A-F, and Table S1). In contrast, blood total B-cell counts were very low in both patients (P2 and P3), with three subsets affected (transitional, naïve, and memory B cells), whereas plasmablast counts were within the range for controls (Figure 6A). The frequency of transitional B cells among the residual B cells was very low, suggesting a central differentiation defect, whereas that of memory B cells was similar to that in controls. In addition, both patients had an abnormally high proportion of age-associated B cells (ABCs), accounting for >30% of naïve B cells (Figure 6B and S7A)^46,47^. These findings were confirmed in our scRNA-seq analysis of PBMCs, and no major differences in gene expression were observed between the B cells of controls and patients (Figure 5F). The B-cell phenotypes were also confirmed in the BM (Figure S5E), except that the proportion of plasmablasts among the patients’ BM mononuclear cells was low (Figure 6C). Consistent with a central production defect, all B-cell maturation steps were severely impaired in the BM of the two patients (Figure 4G, H, and S7B). However, the *IGH* repertoire of sorted blood CD19^+^ B cells from the patients was diverse and without clonal expansion. Furthermore, *V* and *J* gene usage within unique CDR3 sequences was similar to that in the controls (Figure S7C and D), suggesting that the impaired B-cell differentiation was not due to defects in the generation of productive *IGH* rearrangements. Surprisingly, the plasma concentrations of Ig classes and IgG subclasses were all normal, whereas IgA levels were high (Table S1). The patients were seropositive for many pathogens on conventional and Virscan serological testing (Figure S7E and Table S1). Their plasma IgA strongly stained *E. bieneusi* spores in a fluorescence microscopy experiment (Figure S7F). A multiplex serological analysis revealed that P2 and P3 were seropositive for 33 and 29 HPV types, respectively, whereas a group of 20 adult controls displayed seropositivity for a median of six HPV types (range: 1–21) (Figure 6D)^48^. The presence of anti-HPV IgG antibodies, which are T cell-dependent, suggests that anti-HPV immunity is impaired but not abolished in the patients, consistent with the MmuPV1 infection model (Figure S2). Interestingly, allo-hemagglutinin levels against group A were very low in the patients (titers of 1/4 and 1/8 for P2 and P3, respectively), suggesting weak T cell-independent responses (Table S1). Similarly, serological tests for SARS-CoV-2 antibodies remained negative despite vaccination (Sinopharm) in both patients, and documented COVID-19 in P2 (Table S1). Together, these data demonstrate that human FLT3L plays a crucial role in B-cell differentiation and Ab responses, consistent with the recurrent bacterial infections of the lungs observed in patients.

### FLT3L deficiency has a minor impact on NK cell development

We then investigated the impact of FLT3L deficiency on NK cells, a subset severely affected by such deficiency in the mouse model^24,25^. Total blood NK cell counts were normal for P3, but slightly low in P2 (Figure 6E). The frequencies of patients’ CD56^bright^ NK cells, and the distribution of CD56^dim^ NK cell differentiation subsets, based on KIR and NKG2A expression, were normal (Figure 6F and G)^49,50^. In P3, 30% the CD56^dim^ NK cells were NKG2C^+^ CD57^+^, suggesting that patients with FLT3L deficiency can develop adaptive NK cells (Figure 6G). We detected no NKG2C expression in P2 (Figure 6G), who was homozygous for a frequent deletion in the associated gene^51^. However, 40% of P2’s CD56^dim^ NK cells displayed strong CD57 expression and a downregulation of CD161 (Figure 6G), suggesting an adaptive NK cell response to human cytomegalovirus (HCMV), despite *NKG2C* gene deletion^49,52^. Both patients were seropositive for HCMV, but no viral replication was detected by PCR (Table S1). Normal NK cell differentiation was confirmed in our scRNAseq experiment, and by CyTOF immunophenotyping of the patients’ BM samples (Figure 5E, F, and S5E). Together, these data show that human FLT3L deficiency does not impair NK cell differentiation, allowing the normal development of adaptive NK cell responses.

### Normal LC and dermal macrophage levels but a low frequency of dermal DCs

**Figure 7.**
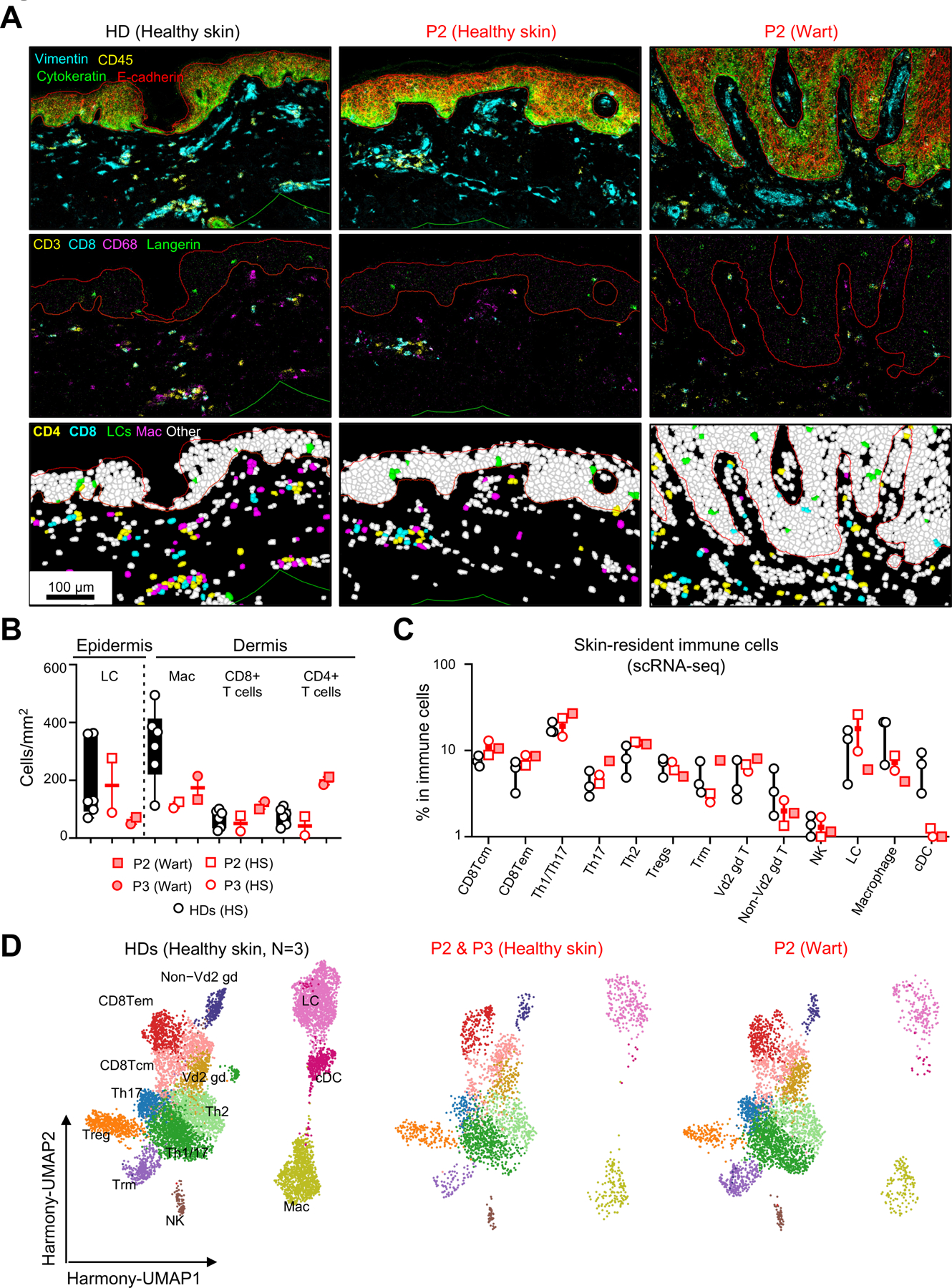
Skin phenotype of FLT3L-deficient patients (A) Imaging mass cytometry (IMC) on paraffin-embedded skin biopsy specimens from HDs or healthy skin (HS) and a wart from P2. In the upper panel, the epidermis and dermis were defined based on cytokeratin and E-cadherin expression. The images in the middle panel show the staining for CD3, CD8, CD68 and langerin. Finally, the images in the lower panel show Langerhans cells (LCs), macrophages (Mac), CD4 and CD8 T cells after segmentation and identification by a machine-learning approach. (B) Quantification of the main immune subsets in the epidermis (LCs) and dermis (Mac, CD4 and CD8 T cells) after identification by a machine-learning approach applied to biopsy specimens from six different HDs (adults, male or female and from multiple ethnic groups), with comparison to HS and warts from two patients. Data are presented as individual values (min to max). Boxplots show the median with IQR. No statistical analysis was performed here. (C) The frequencies of each immune subset after 5′ scRNA-seq on skin biopsy specimens of HS from three HDs (adults, male or female, and from multiple ethnic groups), P2 and P3, and a wart from P2. All the principal clusters targeted are identified on the basis of cell marker expression analysis. Data presentation are as in B. (D) UMAP clustering for the immune subsets shown in 7C.

Finally, the broad susceptibility of the patients to this family of mucocutaneous viruses prompted us to assess the patients’ cutaneous immunity in greater detail. We used imaging mass cytometry and conventional immunohistochemistry to study healthy skin and wart samples from the patients (P2 and P3). We found that the patients’ epidermis contained normal numbers of Langerhans cells (Figure 7A and B), suggesting that the differentiation of these cells was not impaired in the absence of the hematopoietic growth factor FLT3L, consistent with their earlier, yolk sac-derived embryonic origin in mice^40,53^. Langerhans cells were also detected in the epidermis of warts from P2 and P3 (Figure 7A and B). CD4^+^ and CD8^+^ T cells and macrophages were present in normal numbers in healthy dermis from the patients (Figure 7A and B) and were detected below the infected epidermis in the lesions. We increased the cellular resolution, by also performing scRNA-seq on both healthy and infected skin from the patients, after HLA-I^+^CD45^+^ leukocyte enrichment by FACS. With the exception of cDCs, the levels of which were extremely low in the patients, all the leukocyte subsets detected, including Langerhans cells and macrophages, were present at frequencies similar to those in controls (Figure 7C and D). These data suggest that the skin HPV phenotype is not driven by a quantitative decrease in dermal macrophages or epidermal Langerhans cells, instead probably resulting from a loss of dermal dendritic cells.

## Discussion

We describe human FLT3L deficiency, a new inborn error of immunity (IEI) underlying a broad infectious phenotype in three affected siblings. The *FLT3LG* gene was identified in mice and humans in 1993^15^ and 1994^17^, respectively. In FLT3L-deficient mice, the cellularity of the bone marrow is slightly lower than normal^24,25,40^. By contrast, FLT3L-deficient humans have a profoundly hypoplastic bone marrow with an overall decrease in CD34^+^ HSPC counts. The frequencies of megakaryocyte/erythrocyte progenitors (MEPs) among the residual HSPCs of patients are abnormally high, as in the mouse model^54^. The frequency of myeloid progenitors (GMPs) was low in the patients, but unaffected in *Flt3lg*^−/−^ mice^54^. Both humans and mice with FLT3L deficiency display a depletion of DC progenitors^40^. In humans, the proportion of early lymphoid progenitors (MLPs and BNKPs) in the BM of the patients remained within or near the control range, whereas the proportions of B-cell progenitors were low. In mice, the proportion of common lymphoid progenitors (CLPs) and B cell precursors (B-CFU) was remarkably low in the BM of *Flt3lg*^−/−^ mice^24,40,54–56^. Thus, consistent with the model proposed by Tsapogas *et al* based on mouse data^11^, the high proportion of MEPs among FLT3L-deficient HSPCs suggests that the early progenitors not sufficiently stimulated by FLT3L signals preferentially differentiate along the megakaryocyte/erythrocyte pathways. However, this bias allows significant levels of differentiation into most mature hematopoietic subsets, enabling the patients to survive into adulthood, at least with the additional support of modern medicine. The same patients would probably have died at an early age before the advent of vaccination and anti-infectious medicine.

The differences between FLT3L-deficient mice and humans are more apparent in mature hematopoietic cells. Platelet counts were normal in the blood of the FLT3L-deficient patients, but these patients presented slight, probably multifactorial, anemia not observed in the mouse model^24^. As in mice^24^, blood leukocyte cellularity was only moderately low, and neutrophil and total lymphocyte counts were normal or near-normal. Conversely, by contrast to the mouse model^24^, three patients displayed severe blood monocytopenia at most of the time points tested. As in mice^22,23,40,57^, the differentiation of all types of DCs was severely affected in the patients, but not completely abolished, with cDCs and pDCs barely detectable in the blood, BM, and skin. As in mice^24,25^, T-cell counts, including those for αβ and γδ T cells, were normal or near-normal. Accordingly, all *in vitro* APC-independent assays showed T-cell function to be normal. The patients had very few mature circulating B cells (~90% fewer than the controls), and this deficit appeared to be more profound than that in mice (~70%)^24,25^. The residual B cells included very few transitional B cells, and there was an accumulation of ABCs suggestive of an exhaustion of B-cell production^58^. However, like Flt3lg-deficient mice, the patients presented normal immunoglobulin production, and even the production of excessive amounts of IgA (hyper-IgA) despite the low proportion of plasmablasts observed in the BM. Finally, by contrast to Flt3l-deficient mice^24^, the patients had normal or near-normal NK cell counts and differentiation, and displayed normal adaptive NK cell responses to HCMV. Thus, human FLT3L deficiency leads to normal or near-normal platelet, red blood cell, neutrophil and lymphocyte counts, with normal T and near-normal NK cell differentiation, but with a major deficit of B cells and monocytes, and an almost total absence of DCs of all types. The major differences between the patients and the mouse model included profound monocytopenia and near-normal NK cell levels in the patients.

A striking phenotype of the patients was their severe and broad HPV infections. Only a few IEIs underlie skin HPV infections with broad HPV susceptibility and high penetrance. These IEIs include GATA2, CXCR4, and DOCK8 deficiencies, all of which affect the counts or function of T cells and APCs^59,60^. By contrast, FLT3L deficiency does not impair T-cell number or function, but leads to much lower counts of APCs, including circulating monocytes and B cells. The immunophenotyping of skin cells demonstrated the presence of very small numbers of DCs in the patients’ skin, but near-normal and normal counts of macrophages and LCs, respectively. These observations suggest that the patients’ susceptibility to cutaneous viruses is driven principally by an absence of dermal DCs. The absence of reported HPV susceptibility in patients with complete IRF8 deficiency, who lack both monocytes and DCs, is probably due to the death of affected individuals in the absence of hematopoietic stem cell transplantation in early childhood, before exposure to HPV^61–63^. The central role of DCs in immunity to HPV does not exclude a role for LCs or macrophages. Indeed, consistent with the delayed, but not abolished control of MmuPV1 infection in *Flt3lg*^−/−^ mice, the presence of antibodies against a wide range of HPV types in patients (e.g. HPV1, HPV4, HPV6) in the absence of identified related symptoms suggests that the patients develop a T cell-dependent response and can control most HPV infections. However, the characterization of patients with FLT3L deficiency strongly suggests a key non-redundant role for dermal DCs in the optimal control of HPV infections.

The three patients also developed recurrent bacterial infections of the lungs. This phenotype is typical of B-cell defects and consistent with the very low B-cell counts in the patients. Defective B-cell responses despite normal or high Ig levels in the serum are also observed in other IEIs, such as AD STAT3, AD or AR IL6ST, and AR ZNF341 deficiencies^64–68^. FLT3L-deficient patients also display intestinal colonization with *Microsporidium Enterocytozoon bieneusi*, a fungal pathogen generally detected in the context of acquired immunodeficiency syndrome (AIDS)^69,70^. As in FLT3L deficiency, patients with IEIs affecting DC-CD4 crosstalk (HLA-II, CD40, and CD40L deficiencies) have been reported to develop opportunistic fungal infections, due, in particular, to *Microsporidium* and *Cryptosporidium*. This condition can underlie chronic cholangitis and may necessitate liver transplantation in addition to bone marrow transplantion^71–76^. *Microsporidium* colonization of the gut can, therefore, account for the chronic cholangitis in the patients, which, together with *Parechovirus* infection, can account for their chronic diarrhea^77–80^. It is more difficult to explain the polyarthritis reported in P1 and P3. It is tempting to speculate that the defective myeloid differentiation in the patients disrupts the homeostasis of osteoclasts, a cell type responsible for bone resorption that originates from a differentiation pathway common to macrophages^81–83^. The identification and description of more FLT3L-deficient patients and further studies of these patients are now required to clarify the precise mechanism underlying this phenotype.

The patients have a broad infectious phenotype, but the lack of susceptibility to certain types of pathogens is surprising. *Flt3lg*^−/−^ mice presented no unusual susceptibility to natural infections in standard pathogen-free conditions^24^. However, these mice were highly susceptible to *Toxoplasma gondii* and HSV-1^81,84^. In addition, the *Flt3*-deficient *warmflash* mouse strain is susceptible to MCMV^82^. Despite their seropositivity for HSV-1 and HCMV, the two surviving patients did not develop clinical disease. The FLT3L-deficient patients did not develop mycobacterial infections, despite vaccination with BCG and their clear impairment of IL-12 and IFN-γ production *in vitro* in response to BCG stimulation due to the almost total absence of APCs. Susceptibility to mycobacterial infection is a common feature of known DC and monocyte deficiencies^41,61–63,83,85–89^. Similarly, given the rarity of pDCs in the patients, it is surprising that they display no particular susceptibility to respiratory viruses, such as the influenza virus or SARS-CoV-2, which were both documented in P2. Such susceptibility is a common feature of patients with IRF7 and TLR7 deficiencies, both of which impair IFN-α production by pDCs^90–92^. The residual monocytes, together with the rare residual DCs, were probably sufficient to protect the FLT3L-deficient patients against these pathogens. The discovery of new patients will undoubtedly clarify the spectrum of infectious susceptibility in FLT3L-deficient patients. Overall, our findings show that FLT3L plays a key role in human hematopoiesis, with marked differences between FLT3L-deficient patients and *flt3lg*^−/−^ mice, and a surprising viability into adulthood, given the profound multiple cytopenia, including an almost total absence of DCs.

### Limitations of the study

One limitation of our study is that we studied only three relatives from one Iranian kindred. The discovery of other patients, from other kindreds and ancestries, may reveal additional hematological, infectious, or other phenotypes. Another limitation is that we did not study our patients from infancy. The bone marrow phenotype may have been present at birth, or may be a consequence of accelerated senescence, or both.

## STAR★Methods

### Resource availability

#### Lead contact

Further information and requests for resources and reagents should be directed to and will be fulfilled by the lead contact, Vivien Beziat (vivien.beziat@inserm.fr).

#### Materials availability

Under a Material/Data Transfer Agreement (MTA) with INSERM or the Rockefeller University, all raw and processed data as well as biological materials are available upon request from the lead contact.

#### Data and code availability

The single-cell RNA-sequencing data have been deposited at NCBI BioProject and Biostudies EMBL-EBI, and are publicly available as of the publication date. The T cell and B cell receptor datasets have been deposited at Adaptive Biotechnologies. Accession numbers and access links are listed in the key resources table. There is no original code reported in this publication. Upon request, the lead contact will provide and share any more information or row data files needed to reanalyze the data presented in this work.

#### Experimental model and study participant details

The age, sex, ethnicity, ancestry, and race of the studied patients is reported in the main text of the paper and in the method details below. Information on gender and socioeconomic status of the patients and healthy controls was not collected. Written informed consent (IC) was obtained from participants in accordance with local regulations in France, Belgium, the United States of America (USA), and Iran with approval from the appropriate institutional review board (IRB). Experiments were performed in France, Belgium, USA, Iran, and Qatar, in accordance with local regulations and with the approval of the IRB of INSERM and the Rockefeller University for France and the USA, respectively. Patients were included in the C18–41 Genetic Predisposition to Severe Infections study approved by the Sud Est II ethics committee (approval 2022-A00257–36), and the ethics committee of Leuven University Hospital (S62030), and was performed in accordance with the requirements of these bodies. Bone marrow (BM) mononuclear cells were harvested from controls and patients after written, informed consent had been obtained. Control BM cells corresponded to the unused residue of allogeneic hematopoietic stem cells harvested from a healthy adult donor. The patients’ BM cells were obtained from the residual BM after myelogram analysis as part of the diagnostic procedure. The constitution of a collection of BM samples was approved by the regional investigational review board and the French Ministry of Research (references: DC 2011–1338 and DC 2011–1421). The collection was used in accordance with French legislation and the ethical tenets of the Declaration of Helsinki. Virscan studies (on human subjects) were performed with informed written consent, and the procedures were approved by the Institutional Research Ethics Boards of Sidra Medicine and the Qatar Biobank. All work on mice was approved by the Institutional Animal Care and Use Committee of the Pennsylvania State University College of Medicine (PSUCOM), and all procedures were performed in strict accordance with guidelines and regulations.

### Method details

#### Human patients

IC was obtained during the medical examination of the patients in France, Belgium, and residence country of the patients; Iran, in accordance with local regulations and with institutional review board (IRB) approval. A detailed clinical case report is provided below.

#### Case reports

All three patients were siblings born to first-degree consanguineous Iranian parents (Figure 1A, Table S1).

P1 (female) was born in 1989. She suffered from failure to thrive (FTT) from the age of one year. From the age of five years, she presented recurrent infections, such as otitis media leading to hearing impairment, pulmonary infections, and severe disseminated warts (Figure 1B). She also suffered from chronic diarrhea from the age of five years, for which no clear etiology was established at the time. She displayed leukopenia and anemia from the age of seven years. At the age of 10 years, she developed polyarthritis. Tests for antinuclear antibodies and anti-neutrophil cytoplasmic antibodies were negative. P1 died from an undocumented invasive infection after a car accident at the age of 29 years.

P2 (male) was born in 1991. He was healthy until the age of one year, when he presented FTT and chronic diarrhea. Celiac disease was suspected, but the introduction of a gluten-free diet (GFD) led to no improvement. P2 also suffered from early-onset infectious complications: recurrent upper and lower respiratory tract infections (URTI & LRTI) with bilateral hearing impairment due to bilateral otosclerosis at the age of nine years, disseminated common warts from the age of four years (Figure 1B and S1A) and recurrent episodes of oral HSV infection and shingles. He had no adverse reaction to the BCG and MMR live attenuated vaccines. At recent evaluations at the ages of 28 and 31 years, P2 was cachectic and complained of diarrhea and fatigue. Physical examination revealed digital clubbing of the hands and feet, disseminated warts, reduced vesicular lung sounds with expiratory wheezing, and normal cardiac, abdominal and neurological examinations. Chest X ray was normal, and lung computed tomography (CT) revealed bilateral bronchial wall thickening, with no bronchiectasis or lymphadenopathy. P2 repeatedly complained of recurrent diarrhea and weight loss. Abdominal ultrasound revealed dilated intrahepatic biliary ducts; upper GI tract endoscopy revealed normal histological features of the duodenal mucosa; fecal calprotectin and elastase levels were normal; tests for anti-tissue transglutaminase (ATT), anti-endomysial (EMA) and anti-gliadin IgG were negative; IgA-EMA and IgA anti-gliadin tests were negative; IgA-ATT results were weakly positive (37.5 U/mL; normal < 15) in a context of polyclonal IgA hypergammaglobulinemia (38.2 g/L); high loads of microsporidia were observed in the stools (*Encephalitozoon/Enterocytozoon*); HPEV-1 replication rates were high in the stools whereas this virus was only weakly detectable in blood. P2 was diagnosed with chronic cholangitis, possibly due to chronic microsporidiosis (with biliary infection) and plausible chronic HPEV-1 infection. See Table S1 for the detailed laboratory findings.

P3 (female) was born in 1995. Like P2, she presented FTT and diarrhea from the age of one year. Celiac disease was suspected but the introduction of a gluten-free diet (GFD) yielded no improvement, as in P2. P3 also suffered from early-onset infectious complications: recurrent URTI with chronic otorrhea and hearing loss, and recurrent LRTI; HPV infection with disseminated HPV2^+^ common warts from the age of four years (Figure 1B), disseminated HPV5^+^ flat warts (Figure 1B and S1A); recurrent episodes of oral HSV infection; and episodes of *Staphylococcus aureus* cellulitis. P3 displayed no adverse reaction to the BCG and MMR live attenuated vaccines. P3 also presented seronegative idiopathic polyarthritis and Raynaud’s phenomenon from the age of four years, with intermittent swelling affecting the knees, fingers, and shoulders. At recent evaluations at the ages of 24 and 28 years, P3 was cachectic and also complained of diarrhea, chronic cough with yellowish sputum and fatigue. Physical examination found reduced lung vesicular sounds, verrucous plaques and nodules on the hands, feet, lower arms and soles, subungual verruca and HPV7^+^ anogenital warts, with normal cardiac, abdominal and neurological examinations. Chest X ray was normal and lung computed tomography (CT) showed zones of air trapping suggestive of small airway disease (probably constrictive bronchiolitis due to recurrent infections), without bronchiectasis or lymphadenopathy. P3’s chronic diarrhea was investigated further: abdominal ultrasound revealed a dilated bile duct and intrahepatic biliary ducts; upper GI tract endoscopy showed mild neutrophilic duodenitis and no signs suggestive of celiac disease; fecal calprotectin and elastase levels were normal; negative results were obtained in tests for IgG and IgA-ATT, IgG and IgA-EMA, IgA and IgA anti-gliadin; fecal loads of microsporidia were high (*Encephalitozoon/Enterocytozoon*), and high rates of HPEV-1 replication were detected in stools. We concluded that P3 suffered from chronic cholangitis, possibly due to chronic microsporidiosis (with biliary infection) and plausible chronic HPEV-1 infection. See Table S1 for detailed laboratory findings.

#### Whole-exome sequencing (WES) and linkage analysis (LA)

Genomic DNA was isolated from peripheral blood collected from patients, parents, and siblings. WES was performed for P1, P2, P3, their parents and healthy siblings. The DNA preparation was enriched in exons with the SureSelect XT Library prep Kit (Agilent Technologies, CA, USA). Sequencing was performed on an Illumina HiSeq 4000 (San Diego, CA, USA) sequencer with paired-end sequencing, a read length of 101 bp and 100× coverage for the three patients or with a NovaSeq 6000 (Illumina, CA, USA) sequencer with a read length of 150 bp and 100× coverage for the other members of the family, by Macrogen (Macrogen, Inc., Seoul, South Korea). Burrows-Wheeler aligner (BWA) was used to align the reads with the reference genome sequence (GRCh37/hg19)^103^. GATK’s Haplotype Caller from the Genome Analysis Toolkit (GATK version 3.6)^104^ SAMtools^105^, and Picard tools were used for variant calling. All variants were annotated with our in-house annotation software ^26,109,110^. The homozygosity rate was calculated as previously described^111^. We used the WES data for linkage analysis. Assuming a disorder with AR inheritance and complete penetrance, multipoint LOD scores were calculated with MERLIN software^108^.

We focused principally on protein coding sequence-altering variants (nonsense, splice-site variants, coding indels, and missense variants) with minor allele frequencies (MAFs) in the general population reported in the gnomAD database (v2.1.1), and our in-home exome database including >20000 exomes. Variants with high combined annotation-dependent deletion (CADD) (v1.6) scores were filtered out. The mutation significance cutoff (MSC) was calculated as previously described^112^.

#### Sanger sequencing

Genomic DNA samples were extracted from peripheral blood from patients, their parents, and healthy siblings. The region encompassing the *FLT3LG* mutation was amplified by PCR. The PCR products were sequenced by standard Sanger sequencing with the BigDye Terminator Cycle Sequencing Kit (Applied Biosystems). Sequencing reaction products were purified by centrifugation through Sephadex G-50 Superfine resin. Sequencing was performed on an ABI 3500 DNA sequencer (Applied Biosystems, Life Technologies, CA, USA). The primers used for sequencing were those used for PCR. Sequence data were aligned with the genomic sequence of *FLT3LG* (NCBI) with Benchling (https://benchling.com).

#### Metagenomic next-generation sequencing (mNGS)

Urine samples and stools from P2 and P3 were analyzed by metagenomics next-generation sequencing (mNGS) to search for known or unexpected microorganisms. Samples were prepared and sequenced at high sequencing depth in a context of routine mNGS analyses of the patient’s samples, as previously described^113^. Briefly, total nucleic acids were extracted with (stools) or without (urine) prior nuclease treatment. They were then subjected to reverse transcription with random primers and pre-amplification, before production of the final NGS libraries. Sequencing was performed on an Illumina NextSeq500 instrument, generating the following numbers of raw reads: 71.1 M (urine P2), 64.0 M (urine P3), 55.0 M (stools P2), 65.7 M (stools P3). Microseek was used for the detection of microorganisms^114^.

#### Cell culture and isolation

##### Primary cells

Peripheral blood mononuclear cells (PBMCs) were isolated by Ficoll-Hypaque centrifugation (Merck) from cytopheresis products from patients or healthy donors. Cells were either used fresh or were cryopreserved and stored in liquid nitrogen until use. Primary T-cell blasts were generated by culturing PBMCs in ImmunoCult^™^-XF T Cell Expansion Medium (STEMCELL Technologies) supplemented with 10 ng/mL rIL-2 (Thermo Fisher Scientific) (referred to hereafter as T-cell medium or TCM) with replacement of the medium every 48 to 72 hours. Cells were activated with soluble antibody complex reagents (ImmunoCult^™^ Human CD3/CD28/CD2 T-Cell Activator; STEMCELL Technologies) at an initial dilution of 1:80, with reactivation at two-week intervals to ensure continued growth in culture.

##### Cell lines

The HEK293T cell line was purchased from the American Type Culture Collection (ATCC) and cultured in DMEM supplemented with 10% FBS (Gibco). The K562 cell line (kindly donated by Prof. Jerome Tamburini, Institut Cochin) was cultured in RPMI-1640 medium with GlutaMAX (Gibco) supplemented with 10% FBS. EBV-B cell lines were generated in-house by infecting PBMCs with EBV. EBV-B cells were cultured in RPMI-1640 medium with GlutaMAX (Gibco) supplemented with 10% FBS.

#### RNA-seq analyses

We determined the splicing events occurring in different immune cell types by re-analyzing published RNA-seq datasets for human cells (GSE107011)^115^. We downloaded the raw sequence files from the Gene Expression Omnibus (GEO) with the SRA toolkit (fastq-dump), assessed their quality with FastQC (Babraham Bioinformatics), and removed low-quality reads and bases with Trimmomatic v.0.33^116^. The biological replicates for each cell type were aligned with the hg38 reference human genome assembly with HISAT2 v2.2.1^117^ and were combined to obtain greater coverage at exon-splicing junctions. The resulting SAM files were converted to BAM format, sorted, and indexed with samtools v1.12 ^105^. The BAM files were loaded into Integrated Genome Viewer (IGV) to visualize the reads aligned with the *FLT3LG* gene and the spliced reads were counted with the Sashimi plot function ^118^.

#### mRNA extraction, cDNA synthesis, and assessment of gene expression by RT-PCR/RT-qPCR

Total RNA was isolated from the indicated cells with the RNeasy Extraction Kit (QIAGEN) or the Quick-RNA Microprep Kit (Zymo Research Corporation). RNA samples were reverse-transcribed with SuperScript II reverse transcriptase (Thermo Fisher Scientific) and oligo dT primers (Thermo Fisher Scientific) or the High-Capacity RNA-to-cDNA^™^ Kit (Thermo Fisher Scientific), in accordance with the manufacturer’s instructions. RT-qPCR was performed with the TaqMan Gene Expression Master Mix (Applied Biosystems) and specific FAM-MGB probes for *FLT3LG* (Hs00953092_g1 and Hs00181740_m1) (Thermo Fisher Scientific). A human GUSB-VIC probe (4326320E, Thermo Fisher Scientific) was used to normalize the data. RT-qPCR amplification was performed on the Applied Biosystems ViiA 7 system. Relative expression is expressed as 2^−∆Ct^ values after normalization against GUSB (endogenous control) expression (∆Ct).

#### Characterization of *FLT3LG* mRNA splice variants by Topo cloning

Bulk cDNA samples were generated as previous described from the T-cell blasts of P2, his mother, and two unrelated healthy donors. The cDNA from the *FLT3LG* transcripts was amplified with CloneAmp HiFi PCR Premix (Takara) and the following primers binding to exons 1 and 9, respectively, covering the full coding sequence: forward primer: 5’ TTTCGGTCTCTGGCTGTCAC-3’, reverse primer: 5’- CTGTGTCCAGGCTATGCATC-3’. PCR products were inserted into the pCR^™^-Blunt II-TOPO^®^ vector with the Zero blunt TOPO PCR cloning kit (Thermo Fisher Scientific) and were used to transform NEB 10-beta competent *E. coli* cells (New England Biolabs). The bacteria then were spread on LB-agar plates supplemented with kanamycin and incubated overnight at 37°C. Individual colonies were picked, amplified in DreamTaq Green PCR master mix (Thermo Fisher Scientific), and sequenced with the M13 forward and reverse primers from the TOPO cloning kit. The PCR products were subjected to Sanger sequencing as described above and the sequence obtained was aligned with the *FLT3LG* cDNA (NM_001459.4) map with the Benchling application for characterization of the alternative splice variants.

#### Cloning and mutagenesis

For the seven principal transcripts identified, as described above, the full-length WT FLT3L coding sequence (obtained from a cDNA from a healthy donor) was inserted into the pcDNA3.1D plasmid containing a V5 epitope/His by PCR with specific primers for each locus, with the directional TOPO expression kit (Thermo Fisher Scientific). The Tag sequence was located directly after the last coding codon. The patients’ frameshift variant (c.343delC) and the only missense variant reported in the homozygous state in the gnomAD database (https://gnomad.broadinstitute.org/) were constructed by site-directed mutagenesis with CloneAmp Hifi premix (Takara). DpnI (#R0176L, New England Biolabs) was used to digest the resulting PCR products (2 h at 37°C). The product was amplified in NEB 10-beta competent *E. coli* cells (New England Biolabs) and purified with a maxiprep kit (Qiagen) according to the manufacturer’s recommendations. Lentiviral plasmids carrying both the functional *FLT3LG* transcripts (T1 & T7) were inserted into the empty pTRIP-SFFV-deltaNGFR vector (modified from pTRIP-SFFV-mtagBFP-2A; addgene, plasmid #102585) by PCR with modified primers. The empty pTrip-SFFV-ΔNGFR vector was digested with XhoI and BamHI for 1 h at 37°C, and the cDNA of interest, previously amplified by PCR (from pcDNA plasmid), was inserted by homologous recombination with the In-Fusion^®^ HD Cloning Kit, according to the manufacturer’s instructions (#638911, Takara). Each coding sequence was confirmed by Sanger sequencing.

#### Transient transfection

HEK293T cells were used to seed six-well plates at a density of 1 × 10^5^ cells per well and were incubated overnight. The cells were then transiently transfected with the various constructs in the presence of Opti-MEM (Thermo Fisher Scientific) and X-tremeGENE 9 DNA Transfection Reagent (#6365787001, Roche), in accordance with the manufacturers’ instructions, and incubated for 24–72 h before use in various experiments.

#### Cell lysates and analysis of the FLT3L protein by western blotting

Cells were washed with cold PBS and lysed in 100 μL RIPA buffer (50 mM Tris pH 7.4, 150 mM NaCl, 2 mM EDTA, 1% NP-40) supplemented with a mixture of protease and phosphatase inhibitors added to the buffer immediately before use: aprotinin (Sigma, 10 mg/mL), PMSF (Sigma, 1 mM), leupeptin (Sigma, 10 mg/mL), phosSTOP (Roche), and cOmplete Mini Protease Inhibitor Cocktail (Roche). Lysates were incubated for 30 minutes at 4°C and mixed by vortexing every 10 min. The cells were centrifuged for 20 minutes at 16000 x *g* and 4°C, and the supernatant was collected for immunoblotting. Protein yield was determined with the Bradford protein assay (Bio-Rad), and equal amounts of total protein were separated by SDS-PAGE (10% polyacrylamide gel). A recombinant human FLT3L protein (R&D, 308-FKHB-010) was used as positive control. Proteins were transferred onto a polyvinylidene difluoride (PVDF) membrane in a wet transfer system (Bio-Rad). The membrane was blocked by incubation with a blocking buffer (5% BSA or milk in TBS) for 1 h at room temperature. Immunoblotting was performed with the following primary antibodies (overnight incubation at 4°C): anti-Flt 3-L antibody (F-6) HRP (sc-365266 HRP, Santa Cruz), anti-human Flt-3 ligand/FLT3L antibody (MAB308–100, R&D Systems), rabbit anti-FLT3 (8F2) mAb (3462, Cell Signaling), rabbit anti-phospho-FLT3 (Tyr589/591) (30D4) mAb (3464, Cell Signaling), anti-V5 tag antibody (R962–25, Thermo Fisher Scientific) and anti-vinculin antibody (sc-73614 HRP, Santa Cruz). All the primary antibodies were diluted in blocking buffer. For primary Abs not conjugated to HRP, membranes were washed in wash buffer (0.1% Tween 20 in PBS) and incubated with the following secondary antibodies: goat anti-mouse IgG (H + L)-HRP conjugate (1706516, Bio-Rad) or goat anti-rabbit IgG (H + L)-HRP conjugate (1706515, Bio-Rad). Staining was detected with the Clarity Western ECL substrate (Biorad, #1705061) or SuperSignal West Femto (Thermo Fisher Scientific, #34096) with ChemiDoc MP (Biorad). The images were analyzed with Image Lab 6.1 software (Biorad).

#### Lentivirus transduction

HEK293T cells were used to seed a six-well plate at a density of 5 × 10^5^ cells per well, in 2 mL per well DMEM supplemented with 10% FBS, two days before transduction. The cells were then transfected with pCMV-VSV-G (0.2 μg), pHXB2-env (0.2 μg; NIH-AIDS Reagent Program; #1069), psPAX2 (1 μg; a gift from D. Trono; Addgene plasmid #12260) and pTRIP-SFFV-deltaNGFR EV vector or pTRIP-SFFV-deltaNGFR containing the mFL or sFL coding sequences (1.6 μg) in Opti-MEM (Gibco; 300 μl) plus 9 μL X-tremeGENE 9 (Roche), according to the manufacturers’ protocol. After 6 h of transfection, the medium was replaced with 3 mL DMEM supplemented with 10% FBS, and the cells were incubated for a further 24 h to produce lentiviral particles. On the day of HEK293T cell transfection, T-cell blasts from patients and controls were thawed, placed in TCM and reactivated with ImmunoCult^™^ Human CD3/CD28/CD2 T-Cell Activator (STEMCELL Technologies) in a 96-well plate, at a density of 2 × 10^6^ cells/mL in a total volume of 100 μL/well. The next day (day 0), the supernatant of the HEK293T cells was collected and passed through a filter with 0.2 mm pores. Protamine sulfate (Sigma; 10 μg mL^−1^; 8 mg/mL) was added to the viral supernatant, and 100 μL of the supernatant was then added to the activated T cells, which were subjected to spinoculation for 2 hours at 1200 x *g* and room temperature. The spinoculated cells were then incubated for 48 hours at 37°C under an atmosphere containing 5% CO_2_. After two days, the cells were harvested and transferred to small flasks containing TCM supplemented with 1% penicillin/streptomycin.

#### Soluble FLT3L determination

The soluble form of FLT3L was determined in plasma or supernatant collected from cell lines expressing the protein (HEK293T cells and T-cell blasts) by sandwich ELISA (Human Flt-3 Ligand/FLT3L DuoSet ELISA, DY308, R&D Systems) in duplicate and in more than two independent experiments, in accordance with the manufacturer’s protocol. The data obtained were subjected to statistical analysis by the regression analysis approach, in accordance with the instructions supplied with the kit.

#### Analysis of FLT3L expression by flow cytometry

Transiently transfected HEK293T cells were harvested from the wells and washed once with PBS and cold FACS buffer (2% FBS in PBS). The cells were then stained simultaneously with a mixture of Live/Dead Fixable Aqua Dead Cell marker (reconstituted in DMSO; used at a 1:100 dilution in FACS buffer) and a primary Ab against FLT3L (recombinant anti-Flt3 ligand [EP1140Y] (ab52648), Abcam) or an isotype control (recombinant rabbit IgG, monoclonal [EPR25A] - (ab172730), Abcam) antibody for 60 min at 4°C in the dark. The cells were washed three times with FACS buffer and stained with a PE-conjugated goat anti-rabbit IgG (H+L) secondary antibody (A10542, Thermo Fisher Scientific) at a 1:500 dilution, for 30–60 min at 4°C in the dark. Cells were washed twice with FACS buffer and acquired with a Gallios (Beckman Coulter) machine. Data were then analyzed with FlowJo software v10. FLT3L expression on the cell surface of T-cell blasts (non-transduced and transduced) was analyzed with the same staining and analysis procedure.

#### Deep immunophenotyping of whole blood

Deep immunophenotyping was performed by both conventional flow cytometry and mass cytometry (CyTOF) methods. For CyTOF, we used 200 μL fresh whole blood for the patients and controls. We used a custom-designed panel with antibodies indicated in STAR Methods, according to Fluidigm’s instructions. Cells were frozen at −80°C after overnight dead-cell staining and acquisition was performed on a Helios mass cytometer (Fluidigm). All the samples were processed within 24 hours of sampling. Data analysis was performed with OMIQ software.

For classical immunophenotyping of the patients, fresh whole-blood samples were stained by incubation for 30 minutes at room temperature with antibodies against cell-surface markers, indicated in STAR Methods, and analyzed on a BD FACSLyric^™^ flow cytometer.

For a more detailed analysis of DC subsets, more than 10 M (1×10^7^) freshly isolated PBMCs were subjected to cell-surface staining with the following Abs: anti-CD3 FITC (#555332, BD), anti-CD19 FITC (#555412, BD), anti-CD14 FITC (#555397, BD), anti-CD16 FITC (#555406, BD), anti-CD56 FITC (#345811, BD), anti-CD11c APC (#559877, BD), anti-CD45 BV605 (#368523, Biolegend), anti-HLA-DR Pacific Blue (#307633, Biolegend), anti-CD1c APC-Cy7 (#331520, Biolegend), anti-CD141 BV711 (#563155, BD), anti-CD370 (CLEC9A) PE (#353804, Biolegend), anti-CD123 PE-Cy7 (#306010, Biolegend). Staining was performed with the Ab mixture and the Aqua Live/Dead Cell Stain Kit (Thermo Fisher Scientific) for 30 minutes at 4°C. Samples were analyzed on a Fortessa X20 (BD) and data were analyzed with FlowJo 10.8.1 software.

For the analysis of progenitors in blood, we used a published gating strategy^41^. PBMC samples (stored in liquid nitrogen) were thawed and immediately stained with the following Abs: anti-CD3-FITC (#555332, BD), anti-CD19 FITC (#555412, BD), anti-CD14 FITC (#555397, BD), anti-CD16 FITC (#555406, BD), anti-CD56 FITC (#345811, BD), anti-CD34 APC (#343607, Biolegend), anti-CD45RA APC-H7 (#560674, BD), anti-CD123 Pacific Blue (#306044, Biolegend), anti-CD45 BV605 (#368523, Biolegend), anti-CD10 PE (#312204, Biolegend), and anti-CD38 PE-Vio770 (#130–113-428, Miltenyi Biotec).

Staining was performed with the Ab mixture and the Aqua Live/Dead Cell Stain Kit (Thermo Fisher Scientific) for 30 minutes at 4°C. Samples were analyzed on a Fortessa X20 (BD) machine and data were analyzed with FlowJo 10.8.1 software.

#### Bone marrow experiments

Bone marrow mononuclear cells (BMNCs) were isolated by density separation on Lymphoprep (Axis-Shield, Oslo, Norway). CD34^+^ hematopoietic progenitor cells were isolated with an indirect CD34 microbead kit (CD34 MicroBead Kit UltraPure, human, 130–100-453 or CD34 MicroBead Kit, human, 130–046-702) and a magnetic separator (Miltenyi Biotec, Bergisch Gladbach, Germany). The purity of the CD34^+^ cells was checked with a MACSQuant analyzer (Miltenyi Biotec). For the *in vitro* differentiation assays, the ability of the CD34^+^ cells to produce CFUs and BFUs was evaluated in a clonal assay on methylcellulose (MethoCult H04535 and H04435, Stemcell Technologies, Vancouver, Canada) after 12 days of culture, according to the standard protocol. For HSPC phenotyping, the patients and healthy donor cells were characterized with a multi-labeled antibody panel, as listed below. HSPCs were analyzed on a spectral (Sony) flow cytometer, and the data were then analyzed with FlowJo software (v10.8.1). The following Ab panel was used for the HSPC analysis: anti-CD45RA BV711 (#304138, Biolegend), anti-CD90 PE-Cy5 (#328112, Biolegend), anti-CD38 BV605 (#303532, Biolegend), anti-CD34 APC-Cy7 #343514, Biolegend), anti-CD133 PE-Dazzle594 (#372812, Biolegend), anti-CD123 PE-Cy7 (#306010, Biolegend), anti-CD10 BV510 (#312220, Biolegend), anti-CD110 BV421 (#743955, BD) and a Lin custom panel including anti-CD2, anti-CD3, anti-CD4, anti-CD8, anti-CD13, anti-CD14, anti-CD15, anti-CD16, anti-CD19, anti-CD20, anti-CD56, anti-CD235a antibodies, all conjugated with PE and obtained from Miltenyi Biotec.

For the analysis of the B cell precursors by flow cytometry, 200000 isolated BMNCs were used from the patients and one HD for the staining with the following panel: anti-CD45 BV570 (#2120170, Sony), anti-CD20 BV510 (#302339, Biolegend), anti CD34 PE-Cy7 (#2317580, Sony), anti CD19 APC-Cy7 (#2111090, Sony), anti-CD10 APC (#332777, BD), anti-IgM BV421 (#562618, BD), anti-IgD BV711 (#740794, BD), anti-CD24-FITC (#555427, BD), anti-CD38 PE (#555460, BD), and 7AAD (#130–111-568, Miltenyi). Cells were analyzed on an Agilent NovoCyte Flow Cytometers, and the data were then analyzed with FlowJo software (v10.8.1).

For analysis of the leukocyte subsets of the BM, CyTOF was performed on 100 μL fresh whole BM from P2, P3, and the controls, as described above (with the same panel as for blood, described in STAR Methods).

#### Bone biopsy evaluation

Immunostaining was performed on a whole-bone biopsy section in a paraffin block with a standardized Bond Max Leica machine (Leica Microsystems), a Polymer Refine detection kit (Leica), and the following antibodies: anti-glycophorin C (M082001, Agilent), anti-myeloperoxidase (A039829, Agilent) and anti-factor VIII (A008202, Agilent) antibodies.

#### VirScan-phage immunoprecipitation-sequencing (PhIP-Seq)

Antibody profiling by phage immunoprecipitation-sequencing (PhIP-Seq)^119^ and the corresponding data analysis were performed as previously described^98^. Briefly, the experiment was performed on plasma samples from P2, P3, and 20 unrelated controls (representative of the adult general population). We calculated species-specific significance cutoff values to estimate the minimum number of enriched, non-homologous peptides required for a sample to be considered seropositive, as previously described with an in-house dataset and a generalized linear model^119^. The adjusted virus scores were then used to generate heatmap plots. Pooled human plasma used for IVIg (Privigen CSL Behring AG), and human IgG-depleted serum (Molecular Innovations, Inc.) were used as additional controls.

#### Luminex anti-HPV serological tests

Plasma samples from P2, P3, and 20 unrelated healthy controls were sent to the German Cancer Research Center (DKFZ, Heidelberg, Germany) on dry ice for serological analysis. Antibodies against the L1 antigens of HPV types 1, 2, 4, 5, 6, 8, 9, 10, 11, 12, 15, 16, 17, 18, 21, 22, 23, 24, 27b, 31, 33, 36, 38, 41, 45, 48, 50, 52, 58, 60, 75, 80, 88, 92, 93, 96, 101 and 103 were analyzed simultaneously in Luminex-based multiplex serological tests, as previously described^98,120^. In brief, HPV L1 antigens were expressed as recombinant glutathione S-transferase (GST) fusion proteins, loaded onto polystyrene beads and simultaneously presented to primary serum antibodies. The immunocomplexes formed were detected with a biotinylated secondary antibody and streptavidin-R-phycoerythrin as a reporter dye. Serum samples were tested at a dilution of 1:100 and antigen-specific seropositivity was determined on the basis of predefined cutoff values. The values obtained were then used to generate heatmap plots.

#### High-throughput sequencing (HTS) of the human TCR repertoire

DNA was extracted from whole blood from P2, P3, and four unrelated controls with the DNeasy blood & tissue kit (#69504; Qiagen). The rearranged genomic TRB loci were amplified by multiplex PCR on the DNA preparations obtained and sequenced by Adaptive Biotechnologies Seattle, WA. Adaptive Biotechnologies use assay-based and computational techniques to minimize PCR amplification bias. The assay is quantitative, and the frequency of a given TCR sequence is representative of the frequency of the clonotype concerned in the original sample. The PCR products were sequenced on an Illumina HiSeq platform. Custom algorithms were used to filter the raw sequences for errors and to align the sequences with reference genome sequences. The data were then analyzed with ImmunoSeqTM online tools and custom R scripts.

#### High-throughput sequencing (HTS) of the human BCR repertoire

We assessed B-cell receptor (BCR) diversity in these patients with low B-cell counts by performing NGS of the IGH repertoire in sorted circulating B cells. CD19^+^ B cells from P2, P3, and unrelated age-matched healthy control PBMCs were sorted magnetically with anti-CD19 Ab-conjugated microbeads (CD19 MicroBeads, human, 130–050-301, Miltenyi Biotec, Bergisch Gladbach, Germany) according to the manufacturer’s protocol. Isolated B cells were washed with 1X PBS, and DNA was extracted with the DNeasy blood & tissue kit (#69504; Qiagen). The rearranged genomic immunoglobulin heavy chain (IGH) loci were amplified by multiplex PCR on the prepared DNA and sequenced by Adaptive Biotechnologies (Seattle, WA). Adaptive Biotechnologies uses assay-based and computational techniques to minimize PCR amplification bias. The assay was quantitative, and the frequency of a given IGH sequence is representative of the frequency of the clonotype concerned in the original sample. The PCR products were sequenced on an Illumina HiSeq platform. Custom algorithms were used to filter the raw sequences for errors and to align the sequences with reference genome sequences. The data were then analyzed with ImmunoSeqTM online tools, and finally, all analyses were conducted in R v.4 (http://www.R-project.org/) using several custom R scripts.

#### Skin digestion and skin cell sorting

Skin punch biopsies (4 mm) were performed on healthy skin and warts from the patients (P2 and P3) and on healthy skin from three healthy donors. The biopsy specimens were collected in complete RPMI medium (10% FBS and 1% penicillin streptomycin). They were washed with 1X PBS to eliminate the contaminating blood surrounding the tissue and were then incubated in 500 μL 1X dispase (#07923, STEMCELL Technologies) medium in a 24-well plate for 2 h at 37°C, with shaking every 30 minutes. Segments of epidermis were then separated from the dermis. The dermis was cut into chunks with an area of about 1 mm^2^, returned to the same dispase medium and digested overnight (12 h at 37°C) with 500 μL 2X collagenase (#1088866001, Roche). The epidermis was stored in travel medium at 4°C overnight. It was then washed in 1X PBS to eliminate the serum and transferred to a new 24-well plate for digestion for 45 min at 37°C with 500 μL trypsin-EDTA. The epidermal digestion reaction was stopped by adding FBS and the dermal digestion reaction was stopped by adding 6 mM EDTA in PBS (6% FBS). The cells in the digested samples were dissociated by pipetting up and down and the suspension was filtered through a 35 μm-mesh filter. Samples were centrifuged (350 x *g*, 10 min, 4°C) and immediately stained in PBS (supplemented with 0.04% BSA) with anti-CD45 PE (#304008, Biolegend) and anti-HLA-ABC APC (#311409, Biolegend) antibodies and Aqua Live-Dead stain, on ice for 30 min. The cells were then sorted, in microtubes, with a BD FACSAria II sorter (BD Biosciences), into live CD45^+^ and CD45^−^ fractions. Cells from each fraction were then pooled in a specific ratio (20,000 epidermal CD45^−^ cells, all sorted epidermal CD45^+^ cells, 10,000 dermal CD45^−^ cells, and all sorted dermal CD45^+^ cells) and transferred immediately to the 10X Genomics single-cell RNA sequencing platform.

#### Single-cell RNA-sequencing

Single-cell RNA sequencing (scRNA-seq) was performed with different strategies, as explained below. First, to study the bone marrow, we enriched the fresh bone marrow aspirate of two healthy individuals and P2 in CD34^+^ cells as described above in the bone marrow section. Second, for studies of blood leukocyte subsets in the basal state, we used freshly isolated PBMCs from P2, P3, and two healthy donors for library preparation. Third, for enrichment in myeloid cells, we sorted the HLA-DR^+^ monocytes and DCs from the fresh PBMCs of P2, P3, and the two healthy controls (same controls as for the previous experiment on the blood). Cells were stained with a mixture of Abs against cell surface markers and Aqua Live/Dead Cell Stain (same staining as above), and were then sorted in microtubes with a BD FACSAria II sorter (BD Biosciences). Cells were sorted by gating on HLA-DR^+^Lin^−^ (CD3, CD19, CD20, and CD56) cells. The freshly sorted cells were then immediately subjected to RNA sequencing. The Abs used for cell sorting were anti CD3-FITC (#555332, BD), anti-CD19 FITC (#555412, BD), anti-CD20 FITC (#560962, BD), anti-CD56 FITC (#345811, BD), anti-CD11c APC (#559877, BD), anti-HLA-DR PE (#555812, BD), anti-CD1c APC-Cy7 (#331520, Biolegend), anti-CD141 BV711 (#563155, BD), anti-CD123 PE-Cy7 (#306010, Biolegend), anti-CD14 Pacific Blue (#558121, BD), and anti-CD16 BV650 (#563692, BD) antibodies.

Fourth, we used fresh cells obtained from digested biopsy specimens from the healthy skin and lesions of P2 and P3 (as described in the skin digestion section) for scRNA-seq. Library preparation and sequencing were performed in the same manner for all approaches. Briefly, the scRNA-seq libraries were generated with either the Chromium Next GEM Single-Cell 3’ Reagent Kit v3.1 or the Chromium Next GEM Automated Single-Cell 5ʹ Kit v2 with Feature Barcoding (10X Genomics) according to the manufacturer’s protocol. The purified libraries were sequenced on a NovaSeq 6000 (Illumina) machine. We aimed to achieve a median depth of 50000 reads per cell for gene expression.

#### Single-cell RNA-sequencing analysis

The data generated during this study were analyzed in an integrative approach. For the PBMC analysis, we added the data previously obtained for four other controls available in the in-house pipeline of the laboratory (data for four adult controls from different batches of experiments added to the data for the two healthy controls in the experiment), and publicly available control PBMC datasets downloaded from the 10X Genomics web portal (https://support.10xgenomics.com/single-cell-gene-expression/datasets). For the skin analysis, patients were compared with data for four healthy in-house controls acquired at different time points.

Cells were subjected to preprocessing with CellRanger. Approximately 10,000 cells were sequenced per sample. Data were filtered manually with common quality-control metrics and integrated with Harmony^100^. We performed two sequential graph-based clustering analyses. The first-round clustering identified general cell subsets, whereas the second-round clustering identified T-lymphocyte cell subsets at a sufficiently high resolution for analysis. For the first round of clustering, we identified clusters with the SingleR pipeline guided by MonacoImmuneData^115^ for PBMCs and BlueprintEncodeData^121,122^ for skin cells. In the second round of clustering, we used MonacoImmuneData^115^. Cell type-specific marker gene expression was then assessed manually. Clusters were visualized by uniform manifold approximation and projection (UMAP). Gene expression in single cells was quantified with Seurat. Pseudobulk analysis^123^was performed by aggregating all reads from cells assigned to a given cluster, as previously described^106^. Differential expression analysis was performed with DESeq2^107^. P-values were obtained with DEseq2 and the false-discovery rate (FDR) was calculated by the Benjamini-Hochberg procedure; FDR < 0.05 was used as the cutoff. All analyses were conducted in R v.4 (http://www.R-project.org/).

For the BM CD34^+^ HSPCs, data were processed and scRNAseq data were analyzed according to a published pipeline^124^. We filtered on cells expressing CD34 to remove contaminants, due to the low purity of the CD34^+^ cells selected by the magnetic approach for P2 patient. We analyzed a total of 15,164 CD34^+^ cells, with a mean of 14,451 genes detected (SD=143.1). The single-cell RNAseq data are available from Biostudies EMBL-EBI (S-BSST1333). Each individual cell in our dataset was annotated by the Cell-ID method and with reference BM HSPC signatures (*p*<0.01)^42,124^.

#### Whole-blood activation assay

Whole-blood samples from P2, P3 and three healthy donors were collected into heparin-containing collection tubes. Samples were diluted 1:2 in RPMI 1640 supplemented with 100 IU/mL penicillin and 100 μg/mL streptomycin (Thermo Fisher Scientific). Stimulation was performed in a 48-well plate. Briefly, 1 mL of blood was used per well and per treatment. Samples were incubated with medium alone, with live BCG (*M. bovis*-BCG, Pasteur substrain) at a MOI of 20, or with BCG plus IL-12 (20 ng/mL; #219-IL, R&D Systems), BCG plus IL-23 (R&D Systems), or PMA (Merck) for 48 hours at 37°C under an atmosphere containing 5% CO_2_. The supernatants were collected for cytokine determinations. The IFN-γ and TNF contents of the supernatants from the whole-blood stimulation assay were determined in a LEGENDplex^™^ assay (BioLegend) according to the manufacturer’s instructions. Samples were analyzed by flow cytometry on a Gallios flow cytometer. Data were analyzed with LEGENDplex Cloud-based Data Analysis Software (BioLegend).

#### BCG stimulation assay

PBMC samples from P2, P3, healthy donors, and a GATA2-haploinsufficient patient were dispensed into a U-bottomed 96-well plate at a density of 2 × 10^5^ cells per well, in 200 μL lymphocyte medium per well. Cells were incubated in the presence or absence of live BCG, at a multiplicity of infection of 1, with or without recombinant human IL-12 (500 pg/mL, R&D) or recombinant human IL-23 (100 ng/mL, 1290-IL R&D Systems). After 40 hours of stimulation, GolgiPlug (BD Biosciences, 555029; 1:1,000 dilution) was added to each well to inhibit cytokine secretion. After another eight hours of incubation, the cells were collected by centrifugation for flow cytometry analysis. Cells were stained with the Zombie NIR Fixable Viability Kit (BioLegend; 1:2,000 dilution) at room temperature for 15 minutes, and then stained on ice for 30 minutes with a surface-staining panel containing FcR blocking reagent (Miltenyi Biotec; 1:50 dilution), anti-CD3-Alexa Fluor 532 (eBioscience, Clone: UCHT1, 58–0038-42; 1:50 dilution), anti-γδTCR-FITC (eBioscience, Clone:B1.1, 11–9959-41; 1:50 dilution), anti-Vδ2-APC/Fire 750 (BioLegend, Clone:B6, 331419; 1:100 dilution), anti-CD56-BV605 (BioLegend, Clone: 5.1H11, 362537; 1:100 dilution), anti-CD4-BV750 (BD Biosciences, Clone: SK3, 566356; 1:800 dilution), anti-CD8a-Pacific Blue (BioLegend, Clone: SK1, 344717; 1:100 dilution), anti-Vα7.2 TCR-APC (BioLegend, Clone: 3C10, 351708; 1:100 dilution), anti-Vα24-Jα18- PE/Cy7 (BioLegend, Clone: 6B11, 342912; 1:100 dilution), anti-CD20-BV785 (BioLegend, Clone: 2H7, 302356; 1:200 dilution) and anti-PD-1-PE (eBioscience, Clone: MIH4, 12–9969-42; 1:100 dilution) antibodies. Cells were fixed by incubation with 2% paraformaldehyde in PBS on ice for 15 minutes. Cells were then permeabilized/stained by incubation overnight at −20°C in the permeabilization buffer from the Nuclear Transcription Factor Buffer Set (BioLegend), with an intracellular cytokine panel containing FcR blocking reagent (Miltenyi Biotec; 1:50 dilution), anti-IFN-γ-BV711 (BioLegend, Clone: 4 S.B3, 502540; 1:50 dilution), anti-TNF-BV510 (BioLegend, Clone: MAb11, 502950; 1:50 dilution) and anti-IL-10-PE/Dazzle594 (BioLegend, Clone: JES3–19F1, 506812; 1:50 dilution) antibodies. As a positive control, cells in a separate well were stimulated by incubation with PMA (Sigma; 25 ng mL^−1^) and ionomycin (Sigma; 500 nM) for one hour without GolgiPlug followed by a further 7 hours with GolgiPlug (for intracellular cytokine staining). Cells were acquired with an Aurora cytometer (Cytek). Data were analyzed with FlowJo.

#### T-cell proliferation assay

This experiment was performed as for the purposes of diagnosis, in the diagnostic unit of Necker Hospital, Paris (AP-HP).

#### *In-vitro* and *ex-vivo* CD3^+^TCRαβ^+^ CD4^+^ T-cell polarization experiment

Cryopreserved PBMCs were labelled with antibodies directed against CD45RA (BV605, clone HI100, #562886, BD Horizon), and CCR7 (AF700, clone 150503, #561143, BD Pharmingen), CD3 (BV421, clone UCHT1, #562426, BD Horizon), CD8 (BUV395, clone RPA-T8, # 563795, BD Horizon), CD4 (APC, clone RPAT4, #300514, BioLegend), TCRαβ (PECy7, clone IP26, #306720, Biolegend), and TCRγδ (PE, clone B1.1, #12–9959-42, ebioscience). Naïve (defined as CD45RA^+^CCR7^+^) and memory (defined as CD45RA^−^CCR7^+/−^) CD3^+^TCRαβ^+^CD4^+^ T cells were isolated (>98% purity) with a FACS Aria cell sorter (BD Biosciences). Isolated cells were then cultured with T-cell activation and expansion beads (TAE, anti-CD2/CD3/CD28, Miltenyi Biotec) + IL-2 (50 IU/mL, #IL002, Merck) alone (TH0) or under TH1 (IL-12 [50 ng/mL, # 219-IL-005, R&D Systems]), or TH17 (TGF-β1 [2.5 ng/mL, #100–21C-10, Peprotech], IL-1β [50 ng/mL,#200–01B-10, Peprotech], IL-6 [50 ng/mL, #200–06-20, PeproTech], IL-21 [50 ng/mL, #200–21, PeproTech], IL-23 [50 ng/mL, #200–23-10, PeproTech]) polarizing conditions. After five days of polarization, the cells were stimulated with PMA (100 ng/mL, #P8139–1MG, Sigma Aldrich)-ionomycin (750 ng/mL, #I0634–1MG, Sigma Aldrich) for 6 h, with brefeldin A (10 mg/mL, #B7651–5MG, Sigma Aldrich) added after the first 2 h of incubation. For the assessment of intracellular cytokine production, cells were incubated with conjugated monoclonal antibodies directed against IFN-γ (BUV737, clone 4S.B3, #564620, BD Horizon), TNF (PerCP, clone Mab11, #502924, BioLegend), IL-9 (PE, clone MH9A3, #560807, BD Pharmingen), IL-13 (BV421, clone JES10–5A2, #563580, BD Horizon), IL-4 (AF488, clone 8D4–8, #500710, BioLegend), IL-17A (BV510, clone BL168, #512330, BioLegend), IL-17F (BV650, clone O33–782, #564264, BD Horizon), IL-2 (BV750, clone MQ1–17H12, #566361, BD Horizon), IL-21 (eF660, clone eBio3A3-N2, #50–7219-42, Thermo Fisher Scientific), and IL-22 (PerCP e710, clone 22URTI, #46–7229-42, eBioscience). All cells were also stained with the Zombie UV fixable viability dye kit (#423107, Biolegend). Cells and beads were acquired on a BD FACSymphony A5 Cell Analyzer (BD bioscience) and analyzed with FlowJo Software. A previously described gating strategy was used (109).

#### Skin tissue processing and imaging mass cytometry (IMC) data acquisition

We used 3 μm-thick sections of formalin-fixed paraffin-embedded blocks of tissue from cutaneous biopsies performed on patients (warts and normal skin), healthy relatives (normal skin) and healthy donors (normal skin). Slides were first baked for 2 h in a slide oven until all the visible wax had disappeared. They were then dewaxed in a meta-xylene bath for 20 min and rehydrated in a series of descending concentrations of ethanol (100%, 70% and 50%; 10 min each). Slides were washed for five minutes in water and then incubated in antigen retrieval solution for 30 min at 96°C followed by 10 min at room temperature. They were then washed for 10 min in pure water and 10 min in PBS. A PAP pen was used to encircle the samples on each slide, and the slides were then blocked by incubation with 100 μL superblock solution per sample for 45 min in a hydration chamber. Staining was achieved in two steps: a mixture of antibodies diluted in PBS-BSA 0.5% was first applied to the slides, which were then incubated overnight at 4°C in a hydration chamber. The slides were then washed with PBS for 10 minutes and incubated with a second mixture at room temperature for 1.5 h. Our 29-metal antibody IMC panel was designed to evaluate the immune infiltrate present in healthy skin and at the site of infection. It made it possible to study several cell subsets simultaneously, including APCs (DCs, macrophages, monocytes, Langerhans cells), lymphocytes (CD4, CD8, Trm, γδ, Treg, and B cells), neutrophils and keratinocytes (Abs summarized in STAR Methods). Each antibody used in our mixture was previously validated on tonsils, by IMC for conjugated antibodies purchased directly from Fluidigm and by both immunofluorescence and IMC for in-house conjugated antibodies. All conjugations were performed according to the Fluidigm protocol. The stained slides were washed for 16 min in 0.2% Triton-X100 and 16 min in PBS. They were then stained with Intercalator-Ir in Maxpar PBS 1:400 for 30 min at room temperature in a hydration chamber, washed in pure water for 4 min and air-dried for at least 20 min. Finally, IMC data corresponding to pre-established regions of interest (ROIs) were acquired as raw .mcd files with the Hyperion imaging system (Fluidigm).

#### IMC data analysis

IMC data preprocessing and segmentations were performed with the Steinbock toolkit^125^. The preprocessing steps led to the generation of a single .tif file per ROI. Segmentation masks were generated with the deep learning model Deepcell using the iridium channel as the nuclear signal and vimentin, pancytokeratin, CD45, E-cadherin and HLA-DR as cytoplasmic signals. Signal spillover was subsequently compensated as previously described^126^. Segmented and compensated data were then analyzed with Qupath software^127^. In Qupath, cells with an area of 15 μm^2^ were filtered out from downstream analysis. In each remaining cell, various signal data were calculated for each channel (mean, median, minimum and maximum, giving the range, and standard deviation). Based on the vimentin, cytokeratin, CD45, CD4, E-cadherin, CD68, CD8a, CD3 and Langerin channels for an image of healthy skin from a healthy donor, we manually annotated about 10 to 15 cells of each of the following cell subsets: CD4^+^ T cells, CD8^+^ T cells, macrophages, Langerhans cells, other CD45^+^ cells, keratinocytes and other CD45^−^ cells. These annotations were then used to train a random forest cell classifier directly in Qupath. This classifier was then applied to other images with occasional adjustments based on new annotations. In parallel, for quantification, we defined two ROIs in each image: the epidermis, identified with the pancytokeratin and E-cadherin channels, and the 200 μm of subepidermal dermis. This approach enabled us to analyze the composition of the epidermis and the subepidermal dermis accurately for each sample.

#### Mouse papillomavirus (MmuPV1) infection

All mouse work was approved by the Institutional Animal Care and Use Committee of Pennsylvania State University’s College of Medicine (COM), and all methods were performed in accordance with guidelines and regulations. The mice were housed (2–5 mice/cage) in sterile cages within sterile filter hoods in the COM BL2 animal core facility. Infectious mouse papillomavirus (MmuPV1) was isolated from lesions on the tails of mice from our previous studies^128,129^. In brief, papillomas scraped from the tails of the mice were homogenized in phosphate-buffered saline (1×PBS) with a Polytron homogenizer (Brinkman PT10–35) at maximum speed for three minutes, with chilling in an ice bath. The homogenate was spun in a bench centrifuge at 10,000 rpm, the supernatant was decanted into Eppendorf tubes, and the resulting mouse papillomavirus suspension was stored in glycerol at −80°C (1:1,V/V). C57BL/6, *Rag1*^−/−^, and *Flt3lg*^−/−^ (4–6 weeks old) mice were obtained from Jackson Laboratories and reared in the animal core facility of PSUCOM. Three female *Flt3lg*-heterozygous mice in the B6 background were purchased from the Mutant Mouse Resource & Research Centers (MMRRC). Heterozygous female and male mice (from F1 breeding) were crossed to acquire homozygous *Flt3lg*^−/−^ mice, and this knockout was confirmed with the recommended genotyping method. In brief, three mixed primers were used to distinguish between the mutant and wild type: a mutant reverse primer 5’ ATT TGT CAC GTC CTG CAC GAC G 3’; a common primer 5’ TGG CAG CTG AAG TGA CTG AC 3’; and a wild-type reverse primer 5’ AAG CCA AAG CTG GAT GAC AG 3’. We used six female and five male *Flt3lg*^−/−^homozygotes in this study. Mice (4–8 weeks old) were sedated i.p. with 0.1 ml/10 g body weight ketamine/xylazine mixture (100 mg/10 mg in 10 ml double-distilled H_2_O) and their skin was wounded at two sites (tail and muzzle), as previously described^130^. Twenty-four hours after wounding, the mice were again anesthetized and challenged with infectious virus (suspensions of 1×10^9^ viral DNA genomes/ infected site) at the wound sites. Infection was monitored twice weekly at the muzzle and tail wound sites and progress was documented photographically for each animal^128–130^. Two, three, two and four *Flt3lg*^−/−^ mice of both sexes were killed three, four, six and eight weeks, respectively, after viral infection. *Rag1*^−/−^ and C57BL/6 mice were also killed at weeks three, four, six, and eight weeks post infection for comparisons at particular time points. The infected tissues were harvested for additional *in situ* analyses.

#### MmuPV1 DNA copy number analyses

Linearized MmuPV1 genomic DNA was used for standard curve determination with a probe qPCR system. The primer pairs (5’ GGTTGCGTCGGAGAACATATAA 3’ and 5’ CTAAAGCTAACCTGCCACATATC 3’) for E2 amplification were used together with the probe (5’ 6-FAM-TGCCCTTTCA/ZEN/GTGGGTTGAGGACAG 3’-IBFQ). Each reaction contained 9 μL ultrapure water, 5 pmol of each primer, 9 μL Brilliant III qPCR Master Mix (Agilent) and 2 μL DNA template. The PCR conditions were: initial denaturation at 95°C for 3 min, then 40 cycles at 95°C for 5 seconds and 60°C for 10 seconds on an Agilent AriaMx. Viral copy numbers were converted into an equivalent DNA load as follows: 1 ng viral DNA = 1.2 × 10^8^ copies (http://cels.uri.edu/gsc/cndna.html). For the tissue samples, 40 ng total DNA was used for qPCR. All samples were tested at least in duplicate. Viral DNA titers were calculated from a standard curve.

#### Anti-MmuPV1 antibody detection in enzyme-linked immunosorbent assays (ELISA)

Serum was collected from mice at the end of the experiment. ELISA assay was performed for antibodies against MmuPV1 L1. 0.5 μg MmuPV1 L1 VLPs in 50 μL 1X PBS/well was coated in the wells of Nunc^®^ MaxiSorpTM 96-well microtiter plates (Thermo Fisher Scientific, Waltham MA), by incubation at room temperature for 30 minutes. For antibody against MmuPV1 E4 protein, 0.5 μg KLH-conjugated MmuPV1 E4 peptide (PKTTPPRRELFPPTPLTQPP, synthesized by China Peptide)/well in 50 μL bicarbonate (pH 9.6) buffer was incubated overnight with the plates at 37°C. Coated plates were blocked by incubation with PBS/5% dry milk powder for 1 hour at room temperature. ELISA was then performed as previously described^98^. Each of the serum samples was diluted 1:100 or more in PBS/5% dry milk and the dilutions were added to the 96-well plates, which were then incubated for 1 hour at room temperature. Negative controls were either sera collected from non-infected mice or minus primary antibodies. The plates were washed five times with washing buffer (0.05% (v/v) Tween 20 in 1×PBS), and we then added goat anti-mouse IgG (H+L)-alkaline phosphatase (AP) (Southern Biotech) secondary antibody diluted 1:2000 in 5% dry milk powder and incubated the plates for one hour at room temperature. The plates were washed five times with washing buffer, and 100 μL of 1 mg/mL pNPP (para-nitrophenyl phosphate), a substrate for alkaline phosphatase (AP), was added to each well for color development, and absorbance at 405 nm/450 nm was measured with a Fisher Microplate Reader. A mouse monoclonal antibody against MmuPV1L1 (MPV.A4) that neutralizes MmuPV1 infection *in vitro* and *in vivo*^97^ was used as the positive control for L1 detection.

#### *In situ* hybridization (ISH), RNA *in situ* hybridization (RNA-ISH), and immunohistochemistry (IHC) for MmuPV1 detection

Infected tissue biopsy specimens were fixed in 10% neutral buffered formalin and embedded in paraffin. Adjacent sequential sections were cut for hematoxylin and eosin (H&E) staining, *in situ* hybridization (ISH), RNA *in situ* hybridization (RNA-ISH), and immunohistochemistry (IHC), as described in previous studies^94,129,131^. For ISH, a biotin-labeled 3913 bp EcoRV/ BamH1 subgenomic fragment from MmuPV1 was used as the probe for the detection of MmuPV1 DNA in tissues^128^. Access to target DNA was obtained by incubation with 0.2 mg/mL pepsin in 0.1 N HCl at 37°C for 8 min. The sections were washed thoroughly. The biotinylated probe was then applied and the sections were heated at 95°C for 5 min to dissociate the target and probe DNA. Re-annealing was allowed to occur for 2 hours at 37°C. Target-bound biotin was detected with a streptavidin AP conjugate and a colorimetric reaction in BCIP/NBT. For ISH, the nuclei were counterstained with Nuclear Fast Red (Vector Laboratories). Viral RNA was detected in formalin-fixed, paraffin-embedded (FFPE) sections with RNAscope technology (Advanced Cell Diagnostics, Inc.), with custom probes binding to the second exon of the E1^E4 ORF (nt 3139–3419), RNAscope^®^ 2.5 VS Probe- V-MusPV-E4, according to the manufacturer’s protocol. The bound probes were detected by colorimetric staining in the RNAscope 2.5 HD Assay – BROWN, with hematoxylin counterstaining. For IHC, an in-house rabbit anti-MmuPV1 E4 polyclonal antibody (generously provided by Dr. John Doorbar’s laboratory) or an anti-MmuPV1 L1 antibody (an in-house mouse monoclonal antibody, MPV.B9) was used on FFPE sections. The bound antibody was detected with the ImmPRESS anti-rabbit IgG polymer system (Vector # MP-7801) or the M.O.M.^®^ (Mouse on Mouse) ImmPRESS^®^ HRP (peroxidase) Polymer Kit (Vector # MP-2400) with the ImmPACT^®^ NovaRED^®^ Substrate (SK-4805). Before mounting, the slides were counterstained with 50% hematoxylin Gill’s No. 1 solution (Sigma-Aldrich) and 0.02% ammonium hydroxide solution (Sigma-Aldrich)^131^.

#### Immunofluorescent staining of *Enterocytozoon bieneusi*

Indirect immunofluorescence staining was performed on stool smears positive for *Enterocytozoon bieneusi*. Between steps, slides were washed three times in PBS – 0.05 % Tween^®^ 20 (PBS-T). Smears were blocked by overnight incubation at room temperature with 5% (m/v) powdered milk in PBS-T. Serum samples diluted 1/10,000 in 1% powdered milk, 1 % Tween^®^ 20 in PBS were then added and the smears were incubated for 1 h. The smears were then incubated with FITC-conjugated anti-human IgA (Inova Diagnostics, 504045) for 30 minutes. The mounting solution was then added and the slides were examined at x630 magnification under a Leitz fluorescence microscope.

#### Quantification and Statistical Analysis

Descriptive statistics were used for analysis (e.g., median, mean, SD, and minimum and maximum values on plots, as described in the figure legends). Data were analyzed using GraphPad Prism software v.10.1.0 (GraphPad Software). The statistical significance of quantitative differences between groups was assessed in two-tailed unpaired Mann–Whitney *U*-tests, Anova with Bonferroni adjustment, or Wilcoxon rank sum test, as indicated. P values are indicated only for statistically significant comparisons. All data are expressed as the mean ± s.d. calculated from at least three independent experiments unless otherwise stated. For the scRNA-Seq data, pseudobulk principal component analysis and gene-set enrichment analysis were performed as described in the methods.

## Supplementary Material

1 **Table S1**: Biological test results for blood and bone marrow of the patients (P2 and P3), related to Figure 1, 4, 5, 6, S5.

2 Figure S1. Related to Figure 1. Three patients with FLT3L deficiency
(A) Images of the HPV infection phenotype in P2 and P3, showing severe cutaneous warts in two different anatomical areas of the two patients. The common warts on the hands and feet of P2 and P3 were HPV2^+^. Note the numerous HPV5^+^ flat warts on the abdomen of P3, suggesting atypical epidermodysplasia verruciformis (lower right picture)
(B) An analysis of whole-exome sequencing (WES) data identified only seven genes carrying a biallelic rare coding variant within the linked region, assuming recessive inheritance with complete penetrance.
(C) Rare biallelic variants within the linked region. *FLT3LG* was the only one of the seven genes related to the immune system and never reported in the homozygous state in a database of sequences obtained from healthy donors (gnomAD v2.1.1).
(D) The chromatogram shows the Sanger sequencing results for all the patients, and for healthy family members, including heterozygous carriers and two homozygous WT individuals for *FLT3LG*. The site of the mutation is indicated by a dashed red square.

3 Figure S2. Related to Figure 1. FLT3L-deficient mice are susceptible to cutaneous MmuPV1 infections
We used the mouse papillomavirus (MmuPV1) infection model in congenic wild-type (WT) and *flt3lg*^−/−^on C57BL/6 background. The MmuPV1 virus causes skin wart lesions in athymic nude or *Rag1*^−/−^ C57BL/6 mice but not in C57BL/6 WT mice.
(A) Representative images of tail and muzzle lesions (red circles) from RAG1-deficient (middle) and *flt3lg*^−/−^mice (bottom) 3 weeks post infection with MmuPV1. Images of a wild-type B6 mouse (top) are also shown for comparison. We infected the muzzle and tail of 11 (5 males and 6 females) *flt3gl*^−/−^ mice, 6 (3 males and 3 females) *Rag1*^−/−^ mice, and 10 (5 males and 5 females) WT B6 mice with MmuPV1. *Rag1*^−/−^ mice have no T or B cells. We monitored skin lesions between two and eight weeks before the animals were killed. None of the WT C57BL/6 mice developed visible skin lesions, consistent with the findings of previous studies. All three *Rag1*^−/−^ mice developed skin warts three weeks post infection. These lesions persisted until the end of monitoring. *Flt3lg*^−/−^ mice were tested for infection and killed at various time points (weeks 3, 4, and 6) after infection. All *Flt3lg*^−/−^ mice began to develop visible lesions three weeks post infection. The lesions began to regress around week 5 and disappeared during week 6.
(B) Infected tissues from *Flt3lg*^−/−^ mice were harvested at different time points for additional tests, including histology (H&E staining), immunohistochemistry (IHC) and *in situ* hybridization (ISH). Tail tissues from *Flt3lg*^−/−^ mice displayed epidermal hyperplasia, with vacuolated cells and parakeratosis. Strong MmuPV1 DNA and RNA (E1^E4) signals, equivalent to those observed in infected *Rag1*^−/−^ mice, were detected in infected *Flt3lg*^−/−^ mice by *in situ* hybridization three weeks after infection. Using antibodies against E4 and L1, we detected strong viral protein signals in the lesions. Viral signals (DNA/RNA/protein) were absent from the tail tissues of *flt3gl*^−/−^ and WT C57BL/6 mice displaying regression six weeks after viral infection (data not shown). Representative images of a wart and healthy skin from the same FLT3L-deficient animal are shown. The data shown are representative of all the mice tested.
(C) PCR measuring MmuPV1 viral load in the skin lesions of *Flt3lg*^−/−^ and *Rag1*^−/−^ mice 3–6 weeks post infection. Intralesional viral load was determined by qPCR for MmuPV1 E2. In *Flt3lg*^−/−^ mice, the viral DNA load was high in week 3 post infection, began to decrease in week 4 and was undetectable in week 6. In *Rag1*^−/−^ mice, the viral DNA load was high at all tested timepoints.
(D) ELISA determining the levels of anti-MmuPV1 E4 and L1 antibodies in WT or *Flt3lg*^−/−^ mouse plasma sampled 3–8 weeks post infection. Normal anti-MmuPV1 L1 and E4 Ab levels were detected in *Flt3lg*^−/−^. Thus, *Flt3lg*^−/−^ mice can mount an anti-MmuPV1 T- and B-cell response, but their control and clearance of infection are impaired. WT: wild type, Neg.: negative control. Data are presented as individual values (min to max). Boxplots show the median and IQR. Two-tailed Mann–Whitney *U* tests were performed to assess the differences between the negative control, WT and *Flt3gl*^−/−^ mice. *P-value≤0.05, ***P-value<0.001, ****P-value<0.0001, ns: not significant.

4 Figure S3. Related to Figures 2, and 3. *FLT3LG* encodes two functional isoforms
(A) *FLT3LG* mRNA splicing events detected by RNA-seq in human CD8^+^ T cells. Mapping of the coverage of raw sequence reads onto the *FLT3LG* gene is shown, together with the number of reads spliced between exons; only splicing events with more than 2% of the maximum coverage in each cell type are shown. The major exons detected in RNA-seq are depicted and the structures of transcripts T1-T7 are shown; blue rectangles represent exons and connecting lines indicate introns.
(B) The proportion of the indicated *FLT3LG* transcripts obtained by topo cloning on cDNA generated from expanded T-cell blasts from healthy donors (HDs) (*n*=2), P3, and the heterozygous mother (M: mother).
(C) Schematic representation of the 7 principal *FLT3LG* transcripts (>4% of total transcripts) detected by topo cloning on cDNA from a HD. The position of the c.343delC mutation is indicated by a red arrow. The various exons are represented by boxes. The different colors correspond to the different domains of the FLT3L protein. UTR: untranslated region. SP: signal peptide. EC: extracellular domain. TM: transmembrane domain. IC: intracellular domain. Alternative coding sequences (alt. coding seq.) due to frameshifts are defined relative to the canonical isoform (T1/T2).
(D) Proportions of total mRNA corresponding to the *FLT3LG* transcript variants. HEK293T cells were transfected with an empty plasmid or a plasmid containing a sequence corresponding to the indicated *FLT3LG* transcripts. After 24 h, *FLT3LG* mRNA levels were assessed by RT-qPCR. Data are presented as 2^−ΔCt^ values normalized against endogenous GUS control gene expression. The data shown are representative of three independent experiments. NT: not transfected. Bars indicate mean values and the SD.
(E) Cell-surface FLT3L expression as assessed by FACS on HEK293T cells, as in D.
(F) Total FLT3L protein levels on western blots (WB) of protein extracts or culture supernatant from HEK293T cells, as in D and E.
(G) Soluble FLT3L protein determination by ELISA on the supernatant of HEK293T cells, as in D-F. Bars indicate the mean and SD.
(H) Phospho-FLT3 (pFLT3) levels in K562 cells transduced with FLT3, as assessed by WB, after 5 minutes stimulation with the supernatant of HEK293T cells collected 48 h after transfection. A recombinant human FLT3L (rhFLT3L) was used as a positive control. The data shown are representative of three independent experiments.
(I) HEK293T cells were transfected with an empty plasmid or a plasmid containing a sequence encoding the indicated FLT3L transcript. Phospho-FLT3 (pFLT3) levels in K562 cells transduced with FLT3, as assessed by western blotting, after stimulation for 5 minutes in contact with the HEK293T cells 48 h after transfection. A recombinant human FLT3L (rhFLT3L) was used as a positive control.
(J) Proportions of total mRNA corresponding to the *FLT3LG* transcript variants, as assessed by RT-qPCR on mRNA extracted from HEK293T cells 48 hours after transfection with an empty vector (EV), or a vector encoding the WT soluble or membrane-bound FLT3L isoform, or the indicated *FLT3LG* variant. Data are presented as 2^−ΔCt^ values normalized against endogenous *GUS* control gene expression. Bars indicate the mean and SD.
(K) T-cell blasts from patients and controls were either left non-transduced (NT) or were transduced with lentiviruses generated with an empty vector (EV) or with vectors containing the WT sFL or mFL cDNA. Total mRNA was extracted and total *FLT3LG* mRNA levels were determined by RT-qPCR. Data are displayed as 2^−ΔCt^ values normalized against endogenous *GUS* control gene expression. Bars indicate the mean and SD. HD: healthy donor. The data shown are representative of three independent experiments performed.

5 Figure S4. Related to Figure 4. Bone phenotype and bone-marrow HSPC immunophenotyping
(A) Immunostaining on a bone biopsy specimen from P2 showing a predominance of the erythroblastic (glycophorin C (GPC)-positive) and megakaryocytic (factor VIII (FVIII)-positive) lineages, whereas the granulocytic lineage was hypoplastic (myeloperoxidase (MPO)-positive).
(B) Schematic representation of hematopoietic differentiation indicating the progenitor populations: hematopoietic stem cells (HSC), multipotent progenitors (MPP), multilymphoid progenitors (MLP), common myeloid progenitors (CMP), megakaryocyte–erythroid progenitors (MEP), granulocyte–monocyte progenitors (GMP), dendritic cell progenitors (DCP), and B cell–NK progenitors (BNKP).
(C) Gating strategy for the analysis of HSPCs (CD34^+^ cell-enriched) from P2 and one HD.
(D) Gating strategy for the HSPC analysis on the blood of P2, P3 and one HD.
(E) Boxplot representing *FLT3* mRNA levels in the HSPCs and the All-HSC population for each HD (*n*=2) and P2. Wilcoxon rank sum test with continuity correction was used for statistical comparison between the patient and the controls; ***P-value<0.001, **P-value<0.01, ns: not significant.

6 Figure S5. Related to Figures 5 and 6. Blood and bone-marrow immunophenotyping.
(A) Frequency of lymphocytes and neutrophils among blood leukocytes, hemoglobin (HB) levels, and erythrocyte sedimentation rate (ESR) for P1, P2, and P3 at different ages. The reference values for each population are indicated by a gray area.
(B-D) CyTOF performed on whole blood from P2, P3 and 20 HDs (all HDs were adults, male or female, and from multiple ethnic groups). Data are presented as individual values (min to max). Box plots show the median and IQR. Two-tailed Mann–Whitney *U* tests were performed to assess the difference between the patients and controls. The value for a single time point for each individual was used for all statistical analyses. However, the graphs for the patients display different values measured at different timepoints. Significant differences are indicated by asterisks.
(B) Frequency of monocyte subpopulations.
(C) Absolute counts for total T cells and the two main subsets detected by CyTOF.
(D) Absolute counts for all the T-cell subsets detected by CyTOF.
(E) CyTOF performed on total bone-marrow (BM) samples from P2, P3 and four healthy donors (HD). The data show the proportions of the indicated BM leukocyte subsets within the indicated subsets. Data are presented as individual values (min to max). Boxplots indicate the median and IQR. Significant differences are indicated by asterisks.

7 Figure S6. Related to Figure 6. T-cell function and TCR sequencing
(A) CDR3 amino-acid sequence length distributions for the TCR-β locus, based on the sequences of total gDNA extracted from total PBMCs from P2 and P3 in red, and four HDs in gray. No differences were found between patients and controls.
(B) TCR-β clonality and related expansion status. Each colored circle represents a unique TCR sequence, and the area of the circle represents the abundance of that TCR sequence. Black areas (small black circles) represent a TCR repertoire without clonal expansion. No differences were found between patients and controls.
(C) Carboxyfluorescein succinimidyl ester (CFSE) proliferation assay. CD3^+^ T-cell proliferation in response to stimulation with mitogens and antigens. NS = unstimulated, and Stim = stimulated. Red and black indicate the values for patients and healthy donors, respectively. The patients’ cells responded weakly to PHA and had an impaired response to OKT3 and antigenic stimuli (tuberculin, tetanus toxin), probably reflecting the extremely low levels of APCs.
(D) Production of IFNγ and TNF after whole-blood activation with BCG, BCG+IL12, or BCG+IL23, as determined by ELISA. The patients’ cells had an impaired response to BCG, probably reflecting the extremely low levels of APCs
(E) IFNγ production after PBMC stimulation with various stimuli, as assessed by flow cytometry.
(F) Sorted memory (left) or naive (right) CD4^+^ T cells from P2, P3, and healthy donors were either maintained in TH0 conditions (TAE beads) or polarized under TH1 (TAE beads + IL-12) or TH17 (TAE beads + IL-1/IL-6/IL-21/IL-23/TGF-β) conditions. After 5 days of culture, the frequencies of cells positive for the cytokines indicated were assessed by intracellular staining and flow cytometry. Under all polarizing conditions tested, we found no significant difference between the patients’ cells and healthy control cells

8 Figure S7. Related to Figure 6. B-cell phenotype and Virscan assay
(A) CyTOF gating strategy for age-associated B-cell (ABC) subsets in P2, P3, and HDs.
(B) Analysis of B-cell precursors among total bone-marrow mononuclear (BMNCs) cells from P2, P3, and one age-matched HD.
(C) Heatmap illustrating the comparison of *V* and *J* gene usage frequencies in *IGH* productive rearrangements in P2, P3, and four healthy adult controls. The different colors (black to yellow) indicate the different frequencies of gene segment usage from the least (black) to the most frequently used (yellow). ANOVA with Bonferroni adjustment was used for statistical comparison; no significant differences were detected.
(D) Tree maps representing the diversity and clonality of the productive IGH repertoire from P2, P3, and two healthy adult controls. Each dot represents a single CDR3, and the size of the dot represents the relative frequency of that CDR3 in the entire population.
(E) Virscan profiles of the patients, showing the adjusted virus scores for the indicated samples from patients and controls, mock IP samples, IgG-depleted serum, and IVIg. The heatmap shows adjusted virus score values for each sample as a color gradient from blue (antibodies detected but below our significance cutoff values), through purple, to red (for adjusted virus score values above our significance cutoff values). The bar plot (bottom) illustrates the size of the Ab repertoire for a given sample, indicating the precise number of different species displaying peptide enrichment (dark blue) and the number of different species for which the adjusted virus score values exceeded the cutoff values for significance (light blue).
(F) *Enterocytozoon bieneusi* immunofluorescence staining performed on a positive fecal sample with 10,000-fold dilutions of serum from a negative control (not infected), a positive control (infected), P2, and P3. A FITC-coupled anti-IgA antibody was used as secondary antibody. The positive control was slightly positive at this dilution whereas a clear delineated staining of the microsporidial wall was observed with serum from the patients.

## Figures and Tables

**Table:** Key resources table

REAGENT or RESOURCE	SOURCE	IDENTIFIER
**Antibodies**		
anti-Flt 3-L antibody (F-6) HRP	Santa Cruz	sc-365266 HRP
anti-human Flt-3 ligand/FLT3L antibody	R&D Systems	MAB308
rabbit anti-FLT3 (8F2) mAb	Cell Signaling	3462
rabbit anti-phospho-FLT3 (Tyr589/591) (30D4) mAb	Cell Signaling	3464
anti-V5 tag antibody	Thermo Fisher Scientific	R962–25
anti-vinculin antibody HRP	Santa Cruz	sc-73614 HRP
goat anti-mouse IgG (H + L)-HRP conjugate	Bio-Rad	1706516
goat anti-rabbit IgG (H + L)-HRP conjugate	Bio-Rad	1706515
recombinant anti-Flt3 ligand [EP1140Y]	Abcam	ab52648
recombinant rabbit IgG, monoclonal [EPR25A]	Abcam	ab172730
PE-conjugated goat anti-rabbit IgG (H+L) secondary antibody	Thermo Fisher Scientific	A10542
163Dy CXCR3	Fluidigm	3163004B
152Sm TCRgd	Fluidigm	3152008B
142Nd CD19	Fluidigm	3142001B
144 ND CD38	Fluidigm	3144014B
143 ND CD123	Fluidigm	3151001B
153Eu Va7.2	Fluidigm	3153024B
154Sm CD3	Fluidigm	3154003B
155Gd CD45RA	Fluidigm	3155011B
158Gd CD27	Fluidigm	3158010B
159Tb CD1c	Biolegend	331502
161Dy CLEC9A	Fluidigm	3161018B
164Dy CD161	Fluidigm	3164009B
168Er CD8	Fluidigm	3168002B
170Er iNKT	Fluidigm	3170015B
175Lu CCR4	Fluidigm	3175035A
174Yb CD4	Biolegend	300502
162Dy CD21	Miltenyi Biotec Inc.	130–124-315
165Ho NKG2C	Miltenyi Biotec Inc.	130–122-278
148Nd CD20	Biolegend	302302
173Yb HLA-DR	Fluidigm	3173005B
156Gd CCR10	Miltenyi Biotec Inc.	130–122-317
089Y CD45	Fluidigm	3089003B
116Cd CD66b	Biolegend	396902
141Pr CCR6	Fluidigm	3141003A
158Gd CD127	Fluidigm	3143012B
147Sm CD11c	Fluidigm	3147008B
149Sm CD25	Fluidigm	3149010B
150Nd NKVFS1	Bio Rad	MCA2243GA
167Er CCR7	Fluidigm	3167009A
169Tm NKG2A	Fluidigm	3169013B
0171Yb CXCR5	Fluidigm	3171014B
166Er CD24	Fluidigm	3166007B
145ND CD31	Fluidigm	3145004B
160Gd CD14	Fluidigm	3160001B
176Yb CD56	Fluidigm	3176008B
172Yb CD57	Biolegend	359602
150Nd KIR3DL1L2	Miltenyi Biotec Inc.	130–126-489
146Nd IgD	Fluidigm	3146005B
209Bi CD16	Fluidigm	3209002B
CD45 PerCP-Cy5.5	BD	345809
CD19 APC	BD	345791
CD3 FITC	BD	345764
CD16 PE	BD	332779
CD56 PE	BD	345812
CD4 APC	BD	345771
CD8 PE	BD	345773
CD3 PE	BD	345765
CD4 FITC	BD	345768
CD25 APC	BD	340907
CD127 PE	Invitrogen	12–1278-42
CD45 V500	BD	655873
Lin1 FITC	BD	340546
Anti-HLA-DR PerCP	BD	347402
CD123 PE	BD	340545
CD11c APC	BD	333144
CD38 V450	BD	646851
CD19 PE	BD	345777
CD20 PerCP-Cy5.5	BD	345794
CD10 FITC	BD	332775
CD27 APC	BD	337169
CD21 PE	BD	555422
CD3 APC-R700	BD	659119
CD4 APC-H7	BD	641398
CD127 PE-Cy7	BD	560822
CD45RA FITC	BD	335039
CD185 (CXCR5) PerCP-Cy5.5	BD	562781
CD183 (CXCR3) PE	BD	557185
CD196 (CCR6) BV421	BD	562515
BD Pharmingen^™^ FITC Mouse Anti-Human CD3	BD	555332
BD Pharmingen^™^ FITC Mouse Anti-Human CD19	BD	555412
BD Pharmingen^™^ FITC Mouse Anti-Human CD20	BD	560962
BD Pharmingen^™^ FITC Mouse Anti-Human CD14	BD	555397
BD Pharmingen^™^ FITC Mouse Anti-Human CD16	BD	555406
BD^™^ CD56 FITC	BD	345811
APC Mouse Anti-Human CD11C	BD	559877
BD Pharmingen^™^ PE Mouse Anti-Human HLA-DR	BD	555812
BD Pharmingen^™^ Pacific Blue^™^ Mouse Anti-Human CD14	BD	558121
BD Horizon^™^ BV650 Mouse Anti-Human CD16	BD	563692
Brilliant Violet 605^™^ anti-human CD45 Antibody	Biolegend	368523
Pacific Blue^™^ anti-human HLA-DR Antibody	Biolegend	307633
APC/Cyanine7 anti-human CD1c Antibody	Biolegend	331520
BD Horizon^™^ BV711 Mouse Anti-Human CD141	BD	563155
PE anti-human CD370 (CLEC9A/DNGR1) Antibody	Biolegend	353804
PE/Cyanine7 anti-human CD123 Antibody	Biolegend	306010
APC anti-human CD34 Antibody	Biolegend	343607
BD Pharmingen^™^ APC-H7 Mouse Anti-Human CD45RA	BD	560674
Pacific Blue^™^ anti-human CD123 Antibody	Biolegend	306044
PE anti-human CD10 Antibody	Biolegend	312204
CD38 Antibody, anti-human, PE-Vio^®^ 770	Miltenyi Biotec	130–113-428
Brilliant Violet 711^™^ anti-human CD45RA Antibody	Biolegend	304138
PE/Cyanine5 anti-human CD90 (Thy1) Antibody	Biolegend	328112
Brilliant Violet 605^™^ anti-human CD38 Antibody	Biolegend	303532
APC/Cyanine7 anti-human CD34 Antibody	Biolegend	343514
PE/Dazzle^™^ 594 anti-human CD133 Antibody	Biolegend	372812
PE/Cyanine7 anti-human CD123 Antibody	Biolegend	306010
Brilliant Violet 510^™^ anti-human CD10 Antibody	Biolegend	312220
BD OptiBuild^™^ BV421 Mouse Anti-Human CD110	BD	743576
PE conjugated Lin custom panel	Miltenyi Biotec	NA
Brilliant Violet 570^™^ anti-human CD45	Sony	2120170
Brilliant Violet 510^™^ anti-human CD20 Antibody	Biolegend	302339
PE/Cy7 anti-human CD34	Sony	2317580
APC/Cy7 anti-human CD19	Sony	2111090
BD^™^ CD10 APC	BD	332777
BD Horizon^™^ BV421 Mouse Anti-Human IgM	BD	562618
BD OptiBuild^™^ BV711 Mouse Anti-Human IgD	BD	740794
BD Pharmingen^™^ FITC Mouse Anti-Human CD24	BD	555427
BD Pharmingen^™^ PE Mouse Anti-Human CD38	BD	555460
Monoclonal Mouse Anti-Human Glycophorin C (Concentrate)	Agilent	M082001
Polyclonal Rabbit Anti-Human Myeloperoxidase	Agilent	A039829
Polyclonal Rabbit Anti-Human Von Willebrand Factor	Agilent	A008202
PE anti-human CD45 Antibody	Biolegend	304008
APC anti-human HLA-A,B,C Antibody	Biolegend	311409
CD3 Monoclonal Antibody (UCHT1), Alexa Fluor^™^ 532, eBioscience^™^	eBioscience	58–0038-42
TCR gamma/delta Monoclonal Antibody (B1.1), FITC, eBioscience^™^	eBioscience	11–9959-41
APC/Fire^™^ 750 anti-human TCR Vδ2 Antibody	BioLegend	331419
Brilliant Violet 605^™^ anti-human CD56 (NCAM) Antibody	BioLegend	362537
BD Horizon^™^ BV750 Mouse Anti-Human CD4	BD	566356
Pacific Blue^™^ anti-human CD8 Antibody	BioLegend	344717
APC anti-human TCR Vα7.2 Antibody	BioLegend	351708
PE/Cyanine7 anti-human TCR Vα24-Jα18 (iNKT cell) Antibody	BioLegend	342912
Brilliant Violet 785^™^ anti-human CD20 Antibody	BioLegend	302356
CD279 (PD-1) Monoclonal Antibody (MIH4), PE, eBioscience^™^	eBioscience	12–9969-42
Brilliant Violet 711^™^ anti-human IFN-γ Antibody	BioLegend	502540
Brilliant Violet 510^™^ anti-human TNF-α Antibody	BioLegend	502950
PE/Dazzle^™^ 594 anti-human IL-10 Antibody	BioLegend	506812
BD Horizon^™^ BV605 Mouse Anti-Human CD45RA	BD	562886
BD Pharmingen^™^ Alexa Fluor^®^ 700 Mouse anti-Human CD197 (CCR7)	BD	561143
BD Horizon^™^ BV421 Mouse Anti-Human CD3	BD	562426
BD Horizon^™^ BUV395 Mouse Anti-Human CD8	BD	563795
APC anti-human CD4 Antibody	BioLegend	300514
PE/Cyanine7 anti-human TCR α/β Antibody	BioLegend	306720
TCR gamma/delta Monoclonal Antibody (B1.1), PE, eBioscience^™^	eBioscience	12–9959-42
BD Horizon^™^ BUV737 Mouse Anti-Human IFN-γ	BD	564620
PerCP anti-human TNF-α Antibody	BioLegend	502924
BD Pharmingen^™^ PE Mouse Anti-Human IL-9	BD	560807
BD Horizon^™^ BV421 Rat Anti-Human IL-13	BD	563580
Alexa Fluor^®^ 488 anti-human IL-4 Antibody	BioLegend	500710
Brilliant Violet 510^™^ anti-human IL-17A Antibody	BioLegend	512330
BD Horizon^™^ BV650 Mouse Anti-Human IL-17F	BD	564264
BD Horizon^™^ BV750 Rat Anti-Human IL-2	BD	566361
IL-21 Monoclonal Antibody (eBio3A3-N2 (3A3-N2)), eFluor^™^ 660, eBioscience^™^	eBioscience	50–7219-42
IL-22 Monoclonal Antibody (22URTI), PerCP-eFluor^™^ 710, eBioscience^™^	eBioscience	46–7229-42
CD19	Fluidigm	3142014D
Vimentin	Fluidigm	3143027D
CD14	Fluidigm	3144025D
NKp46/NCR1	R&D	MAB1850–100
pan-cytokeratin	Fluidigm	3148022D
CD15	Fluidigm	3149026D
CD134 (OX40)	Fluidigm	3151024D
CD45	Fluidigm	3152018D
TCR-γδ	Santa-cruz	sc-100289
CD11c	Abcam	ab216655
FOXP3	Fluidigm	3155016D
CD4	Fluidigm	3156033D
E-cadherin	Fluidigm	3158029D
CD68	Fluidigm	3159035D
Langerin	Eurobio scientific	DDX0362
CD141	Cell signaling	34149SF
CD8a	Fluidigm	3162035D
CD370/CLEC9A	Abcam	ab245121
CD103	Abcam	ab271889
CD45RA	Fluidigm	3166031D
GZM-B	Fluidigm	3167021D
Ki67	Fluidigm	3168022D
L1	Merck	MAB837
CD3	Fluidigm	3170019D
CD49a	R&D	AF5676
HLA-DR	Fluidigm	3174025D
CD25	Fluidigm	3175036D
CD303	Eurobio scientific	DDX0043
Anti-Human IgA (alpha) FITC	Inova Diagnostics	504045
Antibody against mouse papillomavirus E4	in house	Rabbit serum
Antibody against mouse papillomavirus L1	in house	MPV.B9
Goat Anti-Mouse IgG, Human ads-AP	Southern Biotech	1030–04
Goat Anti-Mouse IgG3, Human ads-AP	Southern Biotech	1100–04
PNPP (p-Nitrophenyl Phosphate, Disodium Sal)	Sigma	487666
**Bacterial and virus strains**		
Mycobacterium bovis BCG	Vogt and Nathan, 2011,^93^ PMID: 21911939	NA
NEB^®^ 10-beta Competent E. coli (High Efficiency)	New England Biolabs	C3019H
NEB^®^ Stable Competent E. coli (High Efficiency)	New England Biolabs	C3040H
Mouse papillomavirus	HSD:NU mouse tail lesions, Hu et al., 2015,^94^ PMID: 26399579	MmuPV1
**Biological samples**		
Plasma from indicated individuals	This manuscript	NA
Peripheral blood mononuclear cells from indicated individuals	This manuscript	NA
Skin Biopsies from indicated individuals	This manuscript	NA
Bone marrow mononuclear cells from indicated individuals	This manuscript	NA
Bone biopsies from indicated individuals	This manuscript	NA
Fresh and FFPE tail and muzzle tissues from Rag1ko, B6, and Flt3lko	Infected tissues from the current study	NA
**Chemicals, peptides, and recombinant proteins**		
ImmunoCult^™^ Human CD3/CD28/CD2 T Cell Activator	STEMCELL Technologies	10970
Gibco^™^ Human IL-2 Recombinant Protein	Thermo Fisher Scientific	PHC0023
Recombinant Human Flt-3 Ligand/FLT3L (HEK293) Protein, CF	R&D	308-FKHB-010
X-tremeGENE^™^ 9 DNA Transfection Reagent	Roche	6365809001
LIVE/DEAD^™^ Fixable Aqua Dead Cell Stain Kit	Thermo Fisher Scientific	L34957
Dispase	STEMCELL Technologies	07923
Collagenase D	Roche	1088866001
Gibco^™^ Trypsin-EDTA (0.05%)	Thermo Fisher Scientific	11580626
GolgiPlug	BD Biosciences	555029
Zombie NIR^™^ Fixable Viability Kit	BioLegend	423105
FcBlock	Miltenyi Biotec	130–059-901
Recombinant human Interleukin-12	R&D	219-IL-025
Recombinant human Interleukin-23	R&D	1290-IL-010
Phorbol 12-myristate 13-acetate	Merck	P1585
Phorbol 12-myristate 13-acetate	Merck	P8139
Interleukin-2 Protein, Recombinant human	Merck	IL002
Recombinant Human TGF-β1	Peprotech	100–21C
Recombinant Human IL-1β	Peprotech	200–01B
Recombinant Human IL-6	Peprotech	200–06
Recombinant Human IL-21	Peprotech	200–21
Recombinant Human IL-23	Peprotech	200–23
Ionomycin calcium salt from Streptomyces conglobatus	Merck	I0634
Zombie UV^™^ Fixable Viability Kit	Biolegend	423107
Brefeldin A	Merck	B7651
KLH-conjugated MmuPV1 E4 peptide (PKTTPPRRELFPPTPLTQPP)	China Peptide	NA
DpnI	New England Biolabs	R0176L
Protamine sulfate	Merck	P3369–10G
Lymphoprep	STEMCELL Technologies	07801
BD Pharmingen^™^ 7-AAD	BD	559925
Cell-ID^™^ Intercalator-Ir	Standard BioTools	201192A
PKTTPPRRELFPPTPLTQPP	Brendle et al., 2023,^95^ PMID: 38133335	Mouse papillomavirus E4 peptide
Mouse papillomavirus L1 virus like particles	Brendle et al., 2021,^96,^ 2022,^97^ PMID: 34578405 PMID: 35920658	MmuPV1L1VLPs
**Critical commercial assays**		
Zero Blunt^™^ TOPO^™^ PCR Cloning Kit	Thermo Fisher Scientific	450245
pcDNA^™^3.1 Directional TOPO^™^ Expression Kit	Thermo Fisher Scientific	K490001
High-Capacity RNA-to-cDNA^™^ Kit	Thermo Fisher Scientific	4387406
SuperScript^™^ II Reverse Transcriptase	Thermo Fisher Scientific	18064014
LEGENDplex^™^ Human Inflammation Panel 1	Biolegend	740809
Human Flt-3 Ligand/FLT3L DuoSet ELISA	R&D	DY308
CD34 MicroBead Kit UltraPure, human	Miltenyi Biotec	130–100-453
CD34 MicroBead Kit, human	Miltenyi Biotec	130–046-702
CD19 MicroBeads, human	Miltenyi Biotec	130–050-301
CD271 MicroBeads	Miltenyi Biotec	130–099-023
T Cell Activation/Expansion Kit, human	Miltenyi Biotec	130–091-441
Chromium Next GEM Single Cell 3’ Reagent Kit v3.1	10X Genomics	CG000315
Chromium Next GEM Single Cell 5’ Reagent Kit v2	10X Genomics	CG000331
SureSelectXT Human All Exon V6	Agilent	5190–8864
Big Dye Terminator v3.1 cycle sequencing kit	Applied Biosystems	4337455
M.O.M.^®^ (Mouse on Mouse) ImmPRESS^®^ HRP (Peroxidase) Polymer Kit	Vector	MP-2400
ImmPRESS anti-rabbit IgG polymer system	Vector	MP-7801
ImmPACT^®^ NovaRED^®^ Substrate	Vector	SK-4805
RNAscope^®^ 2.5 VS Probe- V-MusPV-E4	Advanced Cell Diagnostics, Inc.	473289
RNAscope 2.5 HD Assay BROWN	Advanced Cell Diagnostics, Inc.	322360
Amersham Megaprime DNA Labeling System	Amersham	RPN1604
The Brilliant III qPCR kit	Agilent	600880
The RevertAid First Strand cDNA synthesis kit	Thermo-Fisher	K1622
**Deposited data**		
scRNAseq on fresh PBMCs	This manuscript	NCBI BioProject PRJNA999252
scRNAseq on fresh sorted HLA-DR^+^Lin^−^ (CD3, CD19, CD20, and CD56) cells	This manuscript	NCBI BioProject PRJNA999252
scRNAseq on digested skin biopsies	This manuscript	NCBI BioProject PRJNA999252
scRNAseq on fresh CD34^+^ enriched BMNCs	This manuscript	Biostudies EMBL-EBI (S-BSST1333)
High-throughput sequencing (HTS) of T cell receptor β (TRB) and B cell receptor (IGH) dataset (control and patients)	Adaptive Biotechnologies, This manuscript	https://clients.adaptivebiotech.com/pub/momenilandi-2024-cell (DOI: 10.21417/MM2024C)
**Experimental models: Cell lines**		
HEK293T cells	ATCC	CRL-11268
K562-FLT3	This manuscript	NA
T cell blasts from indicated individuals	This manuscript	NA
EBV-B cells from indicated individuals	This manuscript	NA
**Experimental models: Organisms/strains**		
C57BL/6J	The Jackson Laboratory	https://www.jax.org/strain/000664
C57BL/6-Flt3ltm1Imx/TacMmjax	The Jackson Laboratory	https://www.jax.org/strain/025411 -
B6.129S7-Rag1tm1Mom/J	The Jackson Laboratory, Beziat et al., 2021,^98^ PMID: 34214472	https://www.jax.org/strain/002216
**Oligonucleotides**		
cDNA-FLT3L-Ex1 For 5’ tttcggtctctggctgtcac 3’	Eurofins	NA
cDNA-FLT3L-Ex9 Rev1 5’ ctgtgtccaggctatgcatc 3’	Eurofins	NA
cDNA-topodirect-For 5’ caccatgacagtgctggcgccagc 3’	Eurofins	NA
cDNA-topodirect-Rev1 5’ tcagtgctccacaagcagcaggtcctg 3’	Eurofins	NA
cDNA-topodirect-Rev2 5’ gtgctccacaagcagcaggtcctg 3’	Eurofins	NA
cDNA-topodirect-Rev(c.83)1 5’ tcagtatcctccccaggatgaggcctt 3’	Eurofins	NA
cDNA-topodirect-Rev(c.83)2 5’ gtatcctccccaggatgaggcctt 3’	Eurofins	NA
cDNA-topodirect-Rev(full9c.83)1 5’ tcagtatcctcccctgtaaaatgggatgat 3’	Eurofins	NA
cDNA-topodirect-Rev(full9c.83)2 5’ gtatcctcccctgtaaaatgggatgataga 3’	Eurofins	NA
cDNA-topodirect-Rev(c.121)1 5’ ctatgcatcctctggctggtgactcccctc 3’	Eurofins	NA
cDNA-topodirect-Rev(c.121)2 5’ tgcatcctctggctggtgactcccctc 3’	Eurofins	NA
cDNA-topodirect-Rev(c.260)1 5’ tcatggggacaagggctttgtacagag 3’	Eurofins	NA
cDNA-topodirect-Rev(c.260)2 5’ tggggacaagggctttgtacagag 3’	Eurofins	NA
FLT3L-del7-For 5’ gtgccccccgtccccagtccccaggacctg 3’	Eurofins	NA
Homrec-FLT3L-For 5’ CCAGTACCCTTCACCatgacagtgctggcgccagcct 3’	Eurofins	NA
Homrec-FLT3L-Rev 5’ ctggggacggggggcaccgggctgacactgcagctcca 3’	Eurofins	NA
pcDNA-open-Rev 5’ GGTGAAGGGTACTGGATCCGAGCT 3’	Eurofins	NA
pTrip-dNGFR-For 5’ AGGCACCACCGACAACCTCATCCCT 3’	Eurofins	NA
pTrip-Rev 5’ CTCCTCTTGTGCTTCTAGCCAG 3’	Eurofins	NA
pTrip-FLT3L-For 5’ GAGAACCCTGGACCTatgacagtgctggcgcca 3’	Eurofins	NA
pTrip-FLT3L-Rev1 5’ TTTTCTAGGTCTCGAtcagtgctccacaagcagca 3’	Eurofins	NA
FLT3LG-S22P-For 5’ gctgctgagcccgggactcag 3’	Eurofins	NA
FLT3LG-S22P-Rev 5’ agcagcaggaggagatagg 3’	Eurofins	NA
FLT3LG-S118Afs-For 5’ ccccccccagctgtcttcgctt 3’	Eurofins	NA
FLT3LG-S118Afs-Rev 5’ ctgaaaggcacatttggtgacaaa 3’	Eurofins	NA
FLT3LG-gDNA-For 5’ ATTTCACCACTCCAGCCTGG 3’	Thermo Fisher Scientific	NA
FLT3LG-gDNA-Rev 5’ CCTTTACCGGGCTGACACTG 3’	Thermo Fisher Scientific	NA
Flt3lko mutant reverse 5’ ATT TGT CAC GTC CTG CAC GAC G 3’	Integrated DNA Technologies	NA
Common forward 5’ TGG CAG CTG AAG TGA CTG AC 3’	Integrated DNA Technologies	NA
Flt3l wild type reverse 5’ AAG CCA AAG CTG GAT GAC AG 3’	Integrated DNA Technologies	NA
Forward primers for the mouse papillomavirus 5’-TAGCTTTGTCTGCCCGCACT-3’	Integrated DNA Technologies, Brendle et al., 2021,^96^ PMID: 34578405	NA
Reverse primers for the mouse papillomavirus 5’-GTCAGTGGTGTCGGTGGGAA-3’	Integrated DNA Technologies, Brendle et al., 2021,^96^ PMID: 34578405	NA
Probe for the mouse papillomavirus 5’FAM-CGGCCCGAAGACAACACCGCCACG-3’TAMRA	Integrated DNA Technologies, Brendle et al., 2021,^96^ PMID: 34578405	NA
*FLT3LG*	Thermo Fisher Scientific	Hs00181740_m1
*FLT3LG*	Thermo Fisher Scientific	Hs00953092_g1
*GUSB*	Thermo Fisher Scientific	4326320E
**Recombinant DNA**		
pcDNA3.1/V5-His	Thermo Fisher Scientific	V79020
pCDNA3.1/V5-His-FLT3LG (T1/T2)	This manuscript	NA
pCDNA3.1/V5-His-FLT3LG (T3)	This manuscript	NA
pCDNA3.1/V5-His-FLT3LG (T4)	This manuscript	NA
pCDNA3.1/V5-His-FLT3LG (T5)	This manuscript	NA
pCDNA3.1/V5-His-FLT3LG (T6)	This manuscript	NA
pCDNA3.1/V5-His-FLT3LG (T7)	This manuscript	NA
pCDNA3.1/V5-His-mFLT3LG (T1/2) -Ser118Alafs23*	This manuscript	NA
pCDNA3.1/V5-His-mFLT3LG (T1/2)-Ser22Pro	This manuscript	NA
pCDNA3.1/V5-His-sFLT3LG (T7)-Ser118Alafs23*	This manuscript	NA
pCDNA3.1/V5-His-FLT3LG (T7)-Ser22Pro	This manuscript	NA
psPAX2	Addgene	12260
pCMV-VSV-G	Addgene	8454
pHXB2-Env	NIH-AIDS Reagent Program	1069
pTrip-SFFV-ΔNGFR-2A	In house, Philippot et al., 2023,^99^ PMID: 36763636	NA
pTrip-SFFV-mFLT3LG (T1)-ΔNGFR-2A	This manuscript	NA
pTrip-SFFV-sFLT3LG (T7)-ΔNGFR-2A	This manuscript	NA
The mouse papillomavirus	HQ625439.1 (GenBank) cloned in PUC19	MmuPV1
Mouse papillomavirus L1	Brendle et al., 2021,^96^ PMID: 34578405	MmuPV1 L1
**Software and algorithms**		
Eperico Image scope	Leicabiosystems	https://www.leicabiosystems.com/
R	The R Project for Statistical Computing	https://www.r-project.org/
Harmony	Korsunsky et al., 2019,^100^ PMID: 31740819	https://github.com/immunogenomics/harmony
Cell Ranger	10X Genomics	https://support.10xgenomics.com/single-cell-gene-expression/software/pipelines/latest/what-is-cell-ranger
STAR (2.6.1d)	Dobin et al., 2013,^101^ PMID: 23104886	https://github.com/alexdobin/STAR
Seurat R package	Stuart et al., 2019,^102^ PMID: 31178118	https://cran.r-project.org/web/packages/Seurat/index.html
Burrows-Wheeler aligner	Li et al., 2010,^103^ PMID: 19451168	V0.7.12
GATK	McKenna et al., 2010,^104^ PMID: 20644199	https://www.broadinstitute.org/gatk
SAMtools	Li et al., 2009,^105^ PMID: 19505943	http://samtools.sourceforge.net/
muscat	Crowell et al., 2020,^106^ PMID: 33257685	https://bioconductor.org/packages/release/bioc/html/muscat.html
DESeq2	Love et al., 2014,^107^ PMID: 25516281	https://bioconductor.org/packages/release/bioc/html/DESeq2.html
Picard	http://broadinstitute.github.io/picard/	NA
MERLIN 1.1.2 software	Abecasis et al., 2002,^108^ PMID: 11731797	http://csg.sph.umich.edu/abecasis/merlin/download/
Alamut Visual Plus	https://www.sophiagenetics.com/platform/alamut-visual-plus/	NA
BioRender	https://biorender.com/	NA
**Other**		
		

## Bullet points:

- Human FLT3L deficiency underlies bone-marrow failure and infectious diseases
- Human and mouse FLT3L deficiencies impair dendritic and B-cell development
- FLT3L deficiency impairs monocyte development in humans but not mice
- FLT3L deficiency impairs NK cell development in mice but not humans
